# Takotsubo syndrome diagnosis: the biomarker-based BioTAK score

**DOI:** 10.1093/eurheartj/ehag597

**Published:** 2026-08-31

**Authors:** Florian A Wenzl, Victor Schweiger, Maria A Smolle, Daniel Zech, Alexander Akhmedov, Peizhi Wang, Tetiana Lapikova-Bryhinska, Yujing Wang, Luca Liberale, Karine Santos, Yulia Ilina, Simon Kraler, Andreas Bergmann, Paul Kaufmann, Florian Kahles, Arnold von Eckardstein, Marco Roffi, Lorenz Räber, Victoria L Cammann, Jelena R Ghadri, Davide Di Vece, Thomas F Lüscher, Christian Templin

**Affiliations:** Center for Molecular Cardiology, University of Zurich, Wagistrasse 12, 8952 Schlieren, Switzerland; National Disease Registration Service, NHS England, 10 South Colonnade, Canary Wharf, London E14 4PU, UK; Department of Cardiovascular Sciences, University of Leicester, University Road, Leicester LE1 7RH, UK; Division of Cardiovascular Medicine, Radcliffe Department of Medicine, University of Oxford, John Radcliffe Hospital, Headington, Oxford OX3 9DU, UK; Department of Cardiology, University Hospital Zurich and University of Zurich, Rämistrasse 100, 8091 Zurich, Switzerland; Comprehensive Cancer Center Graz, Medical University of Graz, Auenbrugger Platz 19, 8036 Graz, Austria; Center for Molecular Cardiology, University of Zurich, Wagistrasse 12, 8952 Schlieren, Switzerland; Center for Molecular Cardiology, University of Zurich, Wagistrasse 12, 8952 Schlieren, Switzerland; Center for Molecular Cardiology, University of Zurich, Wagistrasse 12, 8952 Schlieren, Switzerland; Department of Cardiology, State Key Laboratory of Complex Severe and Rare Diseases, Peking Union Medical College Hospital, Beijing, China; Department of Internal Medicine, State Key Laboratory of Complex Severe and Rare Diseases, Peking Union Medical College Hospital, Beijing, China; Center for Molecular Cardiology, University of Zurich, Wagistrasse 12, 8952 Schlieren, Switzerland; Center for Molecular Cardiology, University of Zurich, Wagistrasse 12, 8952 Schlieren, Switzerland; First Clinic of Internal Medicine, Department of Internal Medicine, University of Genoa, Viale Benedetto XV 6, 16132 Genoa, Italy; AOM – IRCCS Ospedale Policlinico San Martino, Largo Rosanna Benzi 19, 16132 Genoa, Italy; 4TEEN4 Pharmaceuticals GmbH, Neuendorfstraße 15B, 16761 Hennigsdorf, Germany; PAM Theragnostics GmbH, Neuendorfstraße 15B, 16761 Hennigsdorf, Germany; Center for Molecular Cardiology, University of Zurich, Wagistrasse 12, 8952 Schlieren, Switzerland; Department of Cardiology, Bern University Hospital, University of Bern, Freiburgstrasse 18, 3010 Bern, Switzerland; PAM Theragnostics GmbH, Neuendorfstraße 15B, 16761 Hennigsdorf, Germany; PAM Theragnostics GmbH, Neuendorfstraße 15B, 16761 Hennigsdorf, Germany; Department of Internal Medicine I, University Hospital Aachen, RWTH Aachen, Pauwelsstraße 30, 52074 Aachen, Germany; Institute of Clinical Chemistry, University Hospital Zurich, Rämistrasse 100, 8091 Zurich, Switzerland; Service de Cardiologie, Hôpitaux Universitaires de Genève, Rue Gabrielle-Perret-Gentil 4, 1205 Geneva, Switzerland; Department of Cardiology, Bern University Hospital, University of Bern, Freiburgstrasse 18, 3010 Bern, Switzerland; Department of Cardiology, University Hospital Zurich and University of Zurich, Rämistrasse 100, 8091 Zurich, Switzerland; Center for Molecular Cardiology, University of Zurich, Wagistrasse 12, 8952 Schlieren, Switzerland; Department of Internal Medicine B, University Medicine Greifswald, Ferdinand-Sauerbruch-Straße, 17475 Greifswald, Germany; SwissCardioVascular Clinic (SwissCVC), Private Hospital Bethanien, Toblerstrasse 51, 8044 Zurich, Switzerland; Department of Internal Medicine B, University Medicine Greifswald, Ferdinand-Sauerbruch-Straße, 17475 Greifswald, Germany; Center for Molecular Cardiology, University of Zurich, Wagistrasse 12, 8952 Schlieren, Switzerland; Heart Division, Royal Brompton and Harefield Hospitals, GSTT, Sydney Street, London SW3 6NP, UK; School of Cardiovascular and Metabolic Medicine, King’s College, Strand, London WCR2 2LS, UK; National Heart and Lung Institute, Imperial College, Guy Scadding Building, Cale Street, London SW3 6LY, UK; Center for Molecular Cardiology, University of Zurich, Wagistrasse 12, 8952 Schlieren, Switzerland; Department of Internal Medicine B, University Medicine Greifswald, Ferdinand-Sauerbruch-Straße, 17475 Greifswald, Germany; SwissCardioVascular Clinic (SwissCVC), Private Hospital Bethanien, Toblerstrasse 51, 8044 Zurich, Switzerland

**Keywords:** Takotsubo syndrome, Acute coronary syndrome, Biomarkers, Machine learning, Diagnosis

## Abstract

**Background and Aims:**

Takotsubo syndrome (TTS) closely mimics acute coronary syndrome (ACS). Early differentiation remains challenging because diagnosis currently relies on medical, psychiatric, and neurological history, repeated cardiac imaging, and exclusion of a culprit epicardial coronary artery lesion, generally with invasive coronary angiography (ICA). The aim of this study was to develop and externally validate a biomarker-based diagnostic score supporting the early differentiation of TTS from ACS before ICA.

**Methods:**

In this study, 3615 patients with ACS or TTS from overlapping registries were investigated. Circulating levels of established and emerging cardiovascular biomarkers were measured before ICA. A biomarker-based diagnostic score for TTS (BioTAK) was developed in a cohort of 1823 patients (ACS *n* = 1754; TTS *n* = 69) using machine learning-guided feature selection and logistic regression and externally validated in an independent cohort of 1792 patients (ACS *n* = 1715; TTS *n* = 77).

**Results:**

The final five-item score incorporated biomarkers reflecting myocardial stress (N-terminal pro-B-type natriuretic peptide), vasoconstriction and anxiety regulation (peptidylglycine α-amidating monooxygenase), atherosclerotic plaque instability (soluble lectin-like oxidized low-density lipoprotein receptor-1), and lipid metabolism (low-density lipoprotein cholesterol), along with sex. BioTAK demonstrated excellent discrimination between TTS and ACS in the development cohort [area under the receiver operating characteristic curve (AUC) .97, 95% confidence interval (CI) .95–.98] and maintained high performance on external validation (AUC .93, 95% CI .90–.96), with good calibration. Using predefined rule-in and rule-out thresholds, the score stratified almost 90% of patients to a likely diagnosis prior to ICA.

**Conclusions:**

TTS is characterized by a distinct biomarker profile reflecting pathophysiological differences from ACS. The non-subjective biomarker-based BioTAK score can support the early identification of TTS, help streamline diagnostic pathways, and might reduce potentially avoidable invasive procedures in patients presenting with suspected ACS.

**Clinical Trial Registration:**

NCT01000701, NCT01947621

## Structured Graphical abstract

**Structured Graphical abstract:**
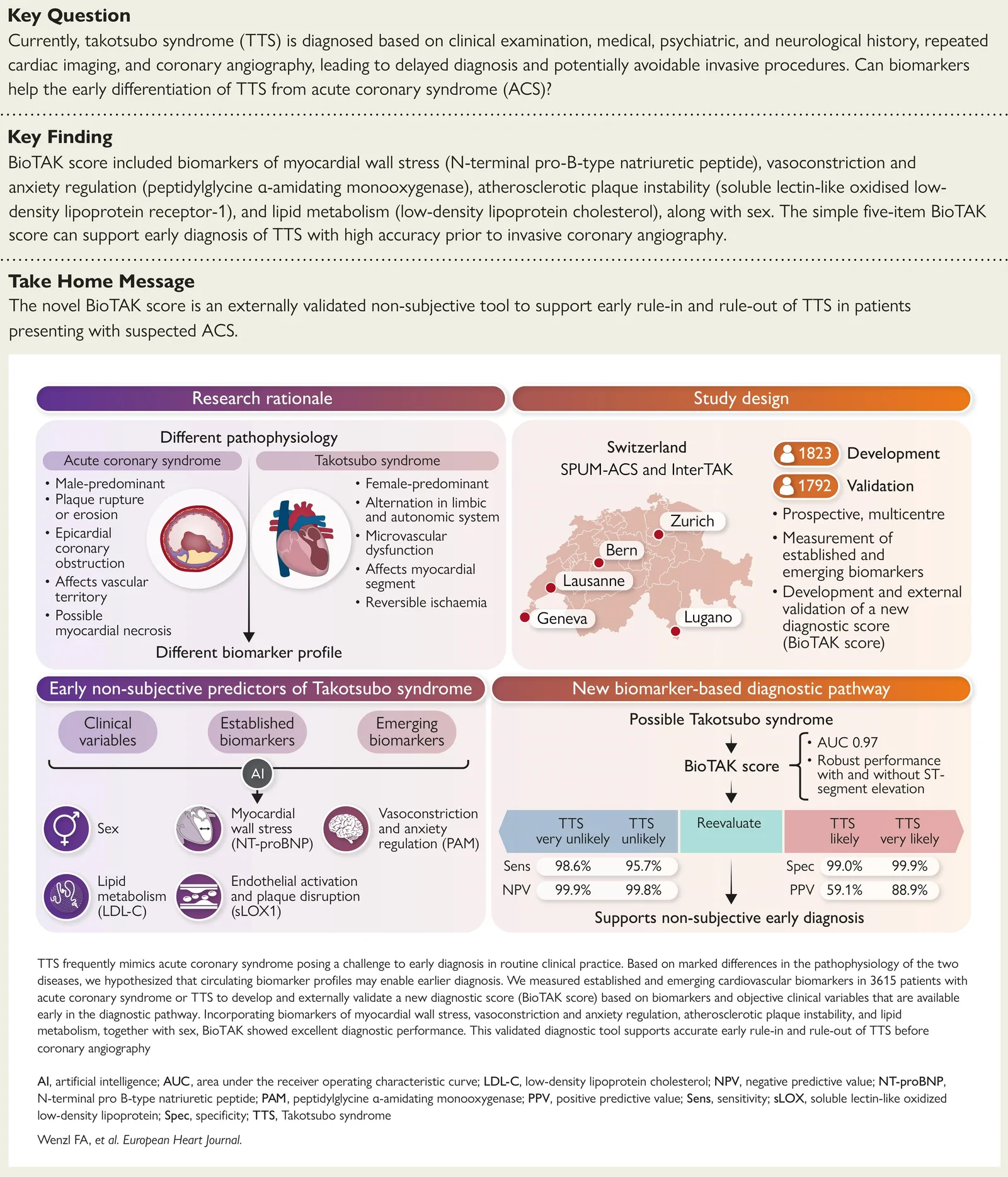

**See the editorial comment for this article ‘Even the most sophisticated cannot escape from the Reverend Bayes', by A.S. Jaffe, https://doi.org/10.1093/eurheartj/ehag656.**

## Introduction

Takotsubo syndrome (TTS) is an acute cardiac condition characterized by transient left ventricular systolic dysfunction and distinctive regional wall motion abnormalities that extend beyond the distribution of a single epicardial coronary artery.^1^ Clinically, patients with TTS present with acute chest pain, electrocardiographic abnormalities, and elevated cardiac biomarkers, closely mimicking acute coronary syndrome (ACS).^1^ TTS is currently diagnosed based on a combination of clinical examination, medical, psychiatric, and neurological history taking, repeated cardiac imaging, and invasive coronary angiography (ICA). Overall, it accounts for ∼1%–4% of patients with suspected ACS.^2–4^

The lack of reliable non-invasive tools for early diagnosis of TTS often necessitates ICA to exclude a culprit epicardial coronary artery lesion. We have previously developed the InterTAK diagnostic score^5^ to support non-invasive diagnosis, however, this tool rests largely on psychiatric and neurological patient history and hinges on identifying or excluding a trigger factor, limiting its objective applicability and practical utility. Established cardiovascular biomarkers appeared promising for supporting the diagnosis of TTS in smaller studies, yet their diagnostic value proved only modest in a larger international validation study.^6^ Likewise, micro ribonucleic acid (miRNA) signatures have shown potential in initial exploratory analyses, but their clinical applicability has remained limited due to high cost, long turnaround time, and technical constraints.^7^ Given the potential consequences of delayed or incorrect diagnosis, diagnostic strategies should combine early applicability within the clinical care pathway with high accuracy, objectivity, and technical feasibility in routine clinical practice.

Recently, several novel biomarkers reflecting key pathophysiological differences between TTS and ACS including myocardial stress, vasoconstriction, anxiety regulation, and endothelial activation have emerged.^8–11^ Here, we sought to develop and externally validate a simple, non-subjective, biomarker-based InterTAK (BioTAK) score for early differentiation of TTS from ACS before ICA.

## Methods

### Study design

Combining two temporally and geographically overlapping prospective registries, we created a large well-characterized cohort of patients with ACS or TTS that features a biobank. We measured circulating levels of established and emerging cardiovascular biomarkers to assess their utility for early diagnosis of TTS. A biomarker-enhanced diagnostic score was developed in consecutive patients treated at University Hospital Zurich, Switzerland between July 2011 and January 2018 and externally validated in patients presenting at a different time and/or at a different study centre. All participants provided written informed consent prior to enrolment. The study was conducted in accordance with the Declaration of Helsinki.

### Participants

We included patients from the SPUM-ACS and InterTAK registries treated between July 2011 and July 2023.

The InterTAK registry (ClinicalTrials.gov ID: NCT01947621; www.takotsubo-registry.com) is an investigator-initiated, multicentre prospective observational study enrolling patients diagnosed with TTS. The design of the InterTAK Registry has been described previously.^12^ Only patients with TTS enrolled at the University Hospital Zurich had available blood samples and were included.

The SPUM-ACS registry (ClinicalTrials.gov ID: NCT01000701) is an investigator-initiated, multicentre prospective observational study including patients presenting with ACS to university hospitals across Switzerland.^8,10,13,14^ At each study site, patient data were documented by a trained study nurse using a web-based centralized data entry system (CARDIOBASE, Clinical Trial Unit and Department of Cardiology, University Hospital Bern, Bern, Switzerland and Webspirit Systems GmbH, Ulm, Germany). Patient recruitment and data collection were overseen by a study committee involving expert cardiologists from each participating study centre.

### Biomarker measurements

Based on the clinical literature and pathobiological plausibility, we studied biomarkers of myocardial stress and haemodynamic disturbance,^10,15–28^ myocardial injury,^1,6,17,29,30^ inflammation,^5,31–37^ plaque instability,^9,38,39^ vasoconstriction and anxiety regulation,^40,41^ renal dysfunction,^8,42–47^ and lipid^48–51^ and glucose metabolism^52^ (see Supplementary data online, *Table S1*). Blood samples were collected at the time of presentation prior to coronary angiography. Following centrifugation, ethylenediaminetetraacetic acid plasma samples were immediately frozen and stored at −80°C in the central biobank at the University Hospital Zurich. Established biomarkers were centrally measured in the core laboratory, University Hospital Zurich, Zurich, Switzerland [high-sensitivity C-reactive protein (hsCRP), high-sensitivity troponin T (hsTnT), and N-terminal pro-B-type natriuretic peptide (NT-proBNP)] or locally in the recruiting hospital. Emerging biomarkers were centrally measured using validated assays (see Supplementary data online, *Table S2*). A detailed description of the biomarkers measured, and the assays used is provided in the Supplementary data online.

### Diagnostic ascertainment

The primary diagnostic outcome was a final diagnosis of TTS. All diagnoses of TTS were established according to the InterTAK diagnostic criteria^53^ at the local study centre and independently confirmed by core members of the InterTAK Study Group. Diagnoses of ACS were made according to pre-specified criteria at the local study centre and reviewed by independent study personnel, as reported previously.^54^

### Score development

Candidate predictors were selected based on their availability before coronary angiography and evidence from the clinical literature (see Supplementary data online). We evaluated bootstrap selection frequency applying machine learning (elastic-net regression) to identify the most reliable predictors for a TTS diagnosis (see Supplementary data online). Based on variable importance (bootstrap selection frequency) and clinical considerations, five non-subjective early predictors were included in a logistic regression model for TTS (see Supplementary data online).^55^ Variables requiring medical history taking were not considered. The final model was transformed into a diagnostic score (i.e. the BioTAK score; 0–60 points) available from a nomogram (see Supplementary data online, *Figure S6*), which assigns integer points to each factor based on the magnitude of association with TTS. The performance of the final model was evaluated in the development and external validation cohorts. Sample size considerations and further information on model development are outlined in the Supplementary data online (*Table S3*).

### Performance evaluation

Model performance was evaluated based on internal validation by calculating bias (optimism)-corrected performance metrics using bootstrap resampling of the development cohort and external validation using unseen data of the validation cohort. We assessed a range of aspects of model performance. Discriminatory performance was evaluated by the area under the receiver operating characteristic curve (AUC). Calibration was evaluated using smoothed calibration curves and by quantifying the calibration slope and the calibration-in-the-large. Clinical utility was evaluated through decision curve analyses (see Supplementary data online). This approach implicitly accounts for both discrimination and calibration considering the ramifications for clinical decision making. For benchmarking, we compared the performance of the BioTAK score to established diagnostic models that require medical history taking or imaging studies and to established biomarker ratios.^5,56^ We conducted several subgroup analyses to assess the robustness of the new score with respect to different population characteristics including age, sex, diabetes, hypertension, dyslipidaemia, presence of ST-segment elevation and evidence of pre-existing coronary artery disease (see Supplementary data online). We tested for non-inferiority of the BioTAK score compared with the established InterTAK diagnostic score,^5^ the diagnostic score proposed by Dagrenat *et al*.,^56^ and established biomarker ratios (NT-proBNP/hsTnT ratio, hsTnT/creatine kinase [CK] ratio, NT-proBNP/CK ratio) at −.05 delta AUC (positive values indicate better performance of BioTAK) using a one-sided hypothesis test (see Supplementary data online).

### Definition and evaluation of diagnostic thresholds

Based on pre-specified performance criteria in the development data [threshold for ruling in TTS: ≥99% specificity and ≥55% positive predictive value (PPV); threshold for ruling out TTS: ≥90% sensitivity and ≥99% negative predictive value (NPV)] we defined groups according to the probability of a TTS diagnosis: a low-probability group (0–26 points; ‘TTS unlikely’), an intermediate probability group (27–32 points; ‘Reevaluate’), and a high-probability group (≥33 points; ‘TTS likely’). In addition, we defined a very-high-probability threshold (≥36 points; ‘TTS very likely’) at ≥99% specificity and ≥80% PPV and a very-low-probability threshold (≤21 points; ‘TTS very unlikely’) at ≥99% sensitivity and ≥99% NPV. These criteria were based on prior studies and clinical considerations.^5^ The diagnostic accuracy of the BioTAK score using these thresholds was assessed by calculating the sensitivity, specificity, PPV, and NPV. Given the varying prevalence of TTS in the literature, we evaluated PPV and NPV across a range of prevalence values (see Supplementary data online, *Table S10*).

### Statistical analyses

A detailed description of the statistical analyses is presented in the Supplementary data online. Continuous variables are presented as mean and standard deviation or as median and interquartile range (IQR), as appropriate. Categorical data are shown as counts and valid percentages. Analyses were conducted using multiply imputed data (20 imputations) and results were pooled using Rubin’s rules, as appropriate (see Supplementary data online, *Table S7*).^8,57,58^ Sensitivity analyses using complete cases were performed to evaluate a potential effect of the imputation on the results (see Supplementary data online, *Table S6*). Data reporting follows the principles outlined by the Transparent Reporting of a multivariable prediction model for Individual Prognosis or Diagnosis (TRIPOD) statement^59^ and the Standards for Reporting of Diagnostic Accuracy Studies (STARD) statement^60^ (see Supplementary data online, *Figures S10* and *S11*). A one-sided alpha level of .025 was used for non-inferiority tests; a two-sided alpha level of .05 was used for all other statistical tests. Confidence intervals (CIs) are two-sided. Data were analysed in R (version 4.3 or later).

## Results

### Characterization of biomarker profiles

We enrolled 1823 patients in the development cohort (ACS *n* = 1754, TTS *n* = 69) and 1792 patients in the validation cohort (ACS *n* = 1715, TTS *n* = 77; see Supplementary data online, *Figure S1*). Patient characteristics are summarized in *Table 1*.

**Table 1:** Baseline characteristics

	Development cohort				Validation cohort			
	Entire cohort (*n* = 1823)	ACS (*n* = 1754)	TTS (*n* = 69)	*P*-value	Entire cohort (*n* = 1792)	ACS (*n* = 1715)	TTS (*n* = 77)	*P*-value
Female sex	402/1823 (22.1)	339/1754 (19.3)	63/69 (91.3)	<.001	430/1792 (24.0)	364/1715 (21.2)	66/77 (85.7)	<.001
Age (years)	64.0 ± 12.6	64.0 ± 12.5	70.5 ± 13.8	<.001	63.8 ± 12.6	63.5 ± 12.5	70.0 ± 14.0	<.001
BMI (kg/m^2^)	26.3 (23.9–29.1)	26.3 (24.0–29.1)	23.4 (20.8–27.1)	<.001	26.8 (24.2–29.7)	26.9 (24.3–29.9)	23.1 (21.9–26.7)	<.001
Systolic blood pressure (mmHg)	131 ± 24	130 ± 23	134 ± 29	.224	127 ± 24	127 ± 24	133 ± 29	.036
Diastolic blood pressure (mmHg)	78 ± 15	78 ± 15	76 ± 14	.478	74 ± 15	74 ± 15	81 ± 19	<.001
Heart rate (bpm)	77 ± 16	77 ± 16	85 ± 22	.001	78 ± 16	77 ± 15	92 ± 21	<.001
ST-segment elevation	1068/1823 (58.6)	1041/1754 (59.4)	27/69 (39.1)	.001	885/1792 (49.4)	862/1715 (50.3)	23/77 (29.9)	<.001
ACS type								
NSTE-ACS	..	762/1754 (43.4)	..		..	877/1715 (51.1)	..	
STEMI	..	992/1754 (56.6)	..		..	838/1715 (48.9)	..	
Takotsubo type	..		..		..		..	
Apical type	..	..	42/69 (60.9)		..	..	45/77 (58.4)¶	
Midventricular type	..	..	21/69 (30.4)		..	..	32/77 (41.6)¶	
Basal type	..	..	3/69 (4.3)		..	..	3/77 (3.9)¶	
Focal type	..	..	3/69 (4.3)		..	..	2/77 (2.6)¶	
Emotional trigger	97/1546 (6.3)	57/1477 (3.9)	40/69 (58.0)	<.001	..	..	36/77 (46.8)	
Physical trigger	238/1546 (15.4)	214/1477 (14.5)	24/69 (34.8)	<.001	..	..	42/77 (54.5)	
Psychiatric disorder	140/1546 (9.1)	117/1477 (7.9)	23/69 (33.3)	<.001	..	..	12/77 (15.6)	
Neurological disorder	158/1546 (10.2)	132/1477 (8.9)	26/69 (37.7)	<.001	..	..	24/77 (31.2)	
ECG								
QTc prolongation	391/1546 (25.3)	370/1477 (25.1)	21/69 (30.4)	.388	..	..	30/77 (39.0)	
Atrial fibrillation	118/1546 (7.6)	113/1477 (7.7)	5/69 (7.2)	1.000	..	..	6/77 (7.8)	
Ischaemia	1474/1698 (86.8)	1432/1629 (87.9)	42/69 (60.9)	<.001	1263/1686 (74.9)	1210/1609 (75.2)	53/77 (68.8)	.261
LVEF (%)	50 (45–60)	51 (45–60)	44 (35–53)	<.001	50 (45–60)	50 (45–65)	41 (35–50)	<.001
Smoking status				<.001				.001
Current smoker	667/1795 (37.2)	649/1726 (37.6)	18/69 (26.1)		667/1765 (37.8)	647/1688 (38.3)	20/77 (26.0)	
Former smoker	498/1795 (27.7)	487/1726 (28.2)	11/69 (15.9)		487/1765 (27.6)	472/1688 (28.0)	15/77 (19.5)	
Never smoker	630/1795 (35.1)	590/1726 (34.2)	40/69 (58.0)		611/1765 (34.6)	569/1688 (33.7)	42/77 (54.5)	
Medical history								
Diabetes	304/1823 (16.7)	293/1754 (16.7)	11/69 (15.9)	.998	310/1792 (17.3)	304/1715 (17.7)	6/77 (7.8)	.036
Hypertension	1035/1823 (56.8)	990/1754 (56.4)	45/69 (65.2)	.187	961/1790 (53.7)	920/1713 (53.7)	41/77 (53.2)	1.000
Dyslipidaemia	1190/1821 (65.3)	1171/1752 (66.8)	19/69 (27.5)	<.001	1062/1792 (59.3)	1032/1715 (60.2)	30/77 (39.0)	<.001
Myocardial infarction	173/1823 (9.5)	170/1754 (9.7)	3/69 (4.3)	.202	219/1785 (12.3)	218/1708 (12.8)	1/77 (1.3)	.005
Cerebrovascular ischaemia	28/1823 (1.5)	21/1754 (1.2)	7/69 (10.1)	<.001	27/1792 (1.5)	23/1715 (1.3)	4/77 (5.2)	.025
Family history of coronary artery disease	407/1780 (22.9)	384/1711 (22.4)	23/69 (33.3)	.049	431/1781 (24.2)	413/1704 (24.2)	18/77 (23.4)	.971
Asthma	53/1546 (3.4)	46/1477 (3.1)	7/69 (10.1)	.005	..	..	5/77 (6.5)	
Malignancy	166/1823 (9.1)	154/1754 (8.8)	12/69 (17.4)	.026	143/1791 (8.0)	131/1714 (7.6)	12/77 (15.6)	.021
Prior PCI	231/1823 (12.7)	225/1754 (12.8)	6/69 (8.7)	.408	277/1792 (15.5)	274/1715 (16.0)	3/77 (3.9)	.007
Prior CABG	56/1823 (3.1)	54/1754 (3.1)	2/69 (2.9)	1.000	55/1792 (3.1)	55/1715 (3.2)	0/77 (.0)	.208
Medication at admission								
Aspirin	471/1208 (39.0)	455/1139 (39.9)	16/69 (23.2)	.008	510/1153 (44.2)	489/1076 (45.4)	21/77 (27.3)	.003
P2Y_12_ inhibitor	98/1208 (8.1)	93/1139 (8.2)	5/69 (7.2)	.965	142/1153 (12.3)	141/1076 (13.1)	1/77 (1.3)	.004
Statin	456/1204 (37.9)	438/1135 (38.6)	18/69 (26.1)	.051	464/1152 (40.3)	449/1075 (41.8)	15/77 (19.5)	<.001
ACE-inhibitor/ARB	679/1199 (56.6)	633/1130 (56.0)	46/69 (66.7)	.108	623/1145 (54.4)	558/1068 (52.2)	65/77 (84.4)	<.001
Beta-blocker	401/1198 (33.5)	382/1129 (33.8)	19/69 (27.5)	.345	403/1148 (35.1)	384/1071 (35.9)	19/77 (24.7)	.063
Calcium channel blocker	242/1198 (20.2)	239/1129 (21.2)	3/69 (4.3)	.001	179/1146 (15.6)	170/1069 (15.9)	9/77 (11.7)	.411
Insulin	77/1208 (6.4)	76/1139 (6.7)	1/69 (1.4)	.141	92/1152 (8.0)	91/1075 (8.5)	1/77 (1.3)	.043
Antidiabetic	198/1208 (16.4)	190/1139 (16.7)	8/69 (11.6)	.347	222/1152 (19.3)	217/1075 (20.2)	5/77 (6.5)	.005
Antidepressant	118/1208 (9.8)	113/1139 (9.9)	5/69 (7.2)	.605	135/1152 (11.7)	125/1075 (11.6)	10/77 (13.0)	.861
Established biomarkers								
eGFR (mL/min/1.73 m^2^)^a^	93 (77–103)	94 (77–103)	81 (62–91)	<.001	91 (75–102)	92 (76–102)	85 (62–94)	<.001
Creatinine (mg/dL)	.86 (.73–1.02)	.87 (.75–1.02)	.84 (.69–1.02)	.139	.88 (.75–1.03)	.88 (.75–1.04)	.78 (.68–.95)	.001
CK (times ULN)	1.2 (.6–2.9)	1.3 (.6–2.9)	.9 (.6–1.2)	.002	.91 (.49–2.09)	.93 (.50–2.15)	.61 (0.41–1.24)	.001
hsTnT (ng/L)	212 (66–628)	216 (66–645)	157 (60–407)	.057	168 (53–546)	160 (52–540)	270 (91–607)	.305
hsCRP (mg/L)	2.4 (1.1–6.5)	2.4 (1.1–6.5)	3.0 (.6–5.7)	.394	2.9 (1.2–7.8)	2.9 (1.2–7.5)	4.0 (1.5–12.0)	.068
NT-proBNP (ng/L)	300 (103–1164)	281 (98–1077)	2369 (384–5748)	<.001	367 (105–1296)	352 (100–1242)	1553 (536–5103)	<.001
Leukocytes (G/L)	9.8 (7.5–12.6)	9.8 (7.5–12.6)	9.3 (7.6–11.5)	.262	9.5 (7.4–11.8)	9.4 (7.4–11.7)	10.4 (8.0–12.9)	.034
Lymphocytes (G/L)	1.6 (1.1–2.1)	1.6 (1.1–2.1)	1.4 (.9–2.0)	.077	1.6 (1.1–2.2)	1.6 (1.1–2.2)	1.5 (.9–2.2)	.170
Neutrophils (G/L)	7.2 (5.1–9.8)	7.2 (5.1–9.8)	6.6 (5.4–9.1)	.349	6.8 (4.9–9.2)	6.8 (4.8–9.1)	7.7 (5.8–10.1)	.018
Neutrophil-lymphocyte ratio	4.5 (2.8–7.3)	4.5 (2.8–7.3)	4.4 (3.2–7.6)	.547	4.3 (2.6–6.8)	4.3 (2.6–6.8)	4.9 (3.0–7.8)	.147
HbA1c (%)	5.7 (5.5–6.1)	5.7 (5.5–6.1)	5.7 (5.5–6.1)	.686	5.7 (5.4–6.4)	5.8 (5.4–6.6)	5.6 (5.3–5.8)	<.001
HDL-C (mg/dL)	45 (37–54)	44 (37–53)	64 (44–74)	<.001	44 (37–54)	44 (34–53)	54 (43–62)	<.001
LDL-C (mg/dL)	118 (90–148)	118 (91–149)	101 (62–124)	<.001	120 (92–148)	120 (93–148)	104 (77–132)	.007
Triglycerides (mg/dL)	81 (55–122)	81 (54–122)	80 (57–116)	.663	97 (66–149)	98 (67–151)	82 (61–132)	.003
Emerging biomarkers								
sLOX-1 (pg/mL)	35 (15–76)	38 (17–79)	4 (2–15)	<.001	31 (13–67)	33 (16–70)	3 (2–7)	<.001
cDPP3 (ng/mL)	20 (16–27)	20 (16–27)	19 (14–28)	.480	18 (14–26)	18 (14–26)	18 (15–26)	.471
PENK (pmol/L)	64 (50–81)	63 (50–81)	66 (50–80)	.909	62 (49–80)	62 (50–80)	61 (50–81)	.525
PAM (ng/mL)	72 (63–83)	72 (62–82)	87 (79–98)	<.001	73 (62–84)	73 (62–83)	83 (74–96)	<.001
ADM (pg/mL)	22 (16–30)	22 (16–30)	21 (14–30)	.542	20 (15–29)	20 (15–28)	21 (15–39)	.241
ADM-Gly (pg/mL)	33 (24–49)	33 (24–48)	41 (24–71)	.020	30 (21–42)	30 (21–41)	40 (27–63)	<.001

Categorical data are shown as *n/*N (%). Continuous data are either presented as mean ± standard deviation or median (interquartile range). Groups were compared using unpaired *t*-tests for normally distributed variables, Mann–Whitney *U* test for non-normally distributed variables and χ^2^ test for categorical variables.

ACE, angiotensin-converting enzyme; ACS, acute coronary syndrome; ADM, adrenomedullin; ADM-Gly, glycine-extended adrenomedullin; ARB, angiotensin II receptor blocker; BMI, body mass index; CABG, coronary artery bypass grafting; cDPP3, circulating dipeptidyl peptidase 3; CK, creatine kinase; ECG, electrocardiogram; HbA1c, glycated haemoglobin; HDL-C, high-density lipoprotein cholesterol; hsCRP, high-sensitivity C-reactive protein; hsTnT, high-sensitivity troponin T; LDL-C, low-density lipoprotein cholesterol; LVEF, left ventricular ejection fraction; NSTE, non-ST-elevation; NT-proBNP, N-terminal pro-B-type natriuretic peptide; PAM, peptidylglycine α-amidating monooxygenase; PENK, proenkephalin; PCI, percutaneous coronary intervention; sLOX-1, soluble lectin-like oxidized low-density lipoprotein receptor-1; STEMI, ST-elevation myocardial infarction; TTS, takotsubo syndrome; ULN, upper limit of normal. ¶ In several patients, more than one type of takotsubo syndrome was recorded.

^a^The eGFR was calculated using the Chronic Kidney Disease Epidemiology Collaboration (CKD-EPI) 2021 equation.^61^

Both patients with ACS and those with TTS displayed signs of myocardial injury. While hsTnT levels were similar in ACS and TTS, creatine kinase (CK) was markedly higher in patients with ACS (see Supplementary data online, *Figure S2*). Patients with TTS displayed more severe cardiac wall stress, witnessed by higher levels of NT-proBNP and glycine-extended adrenomedullin (ADM-Gly). Markers of systemic inflammation, such as hsCRP and neutrophil-lymphocyte ratio, were largely comparable in ACS and TTS. Patients with TTS showed a more favourable lipid profile evidenced by lower levels of low-density lipoprotein cholesterol (LDL-C) and higher levels of high-density lipoprotein cholesterol (HDL-C). Soluble lectin-like oxidized low-density lipoprotein receptor-1 (sLOX-1), a marker of endothelial activation and plaque disruption, was markedly higher in ACS than in TTS. Circulating levels of peptidylglycine α-amidating monooxygenase (PAM), an enzyme involved in sympathetic activation and anxiety regulation, were higher in TTS compared with ACS. Renal function was reduced in TTS compared with ACS, as indicated by lower estimated glomerular filtration rate (eGFR). In the context of higher age and a higher proportion of female patients, creatinine levels were not elevated in TTS compared with ACS.

### Diagnostic value of established and emerging biomarkers

Data-driven variable importance analysis ranked seven biomarkers among the top 15 variables in terms of diagnostic value, of which sLOX-1 and PAM had the highest predictive importance (*Figure 1*). Other important biomarkers included markers of myocardial stress (NT-proBNP), lipid metabolism (LDL-C, HDL-C), inflammation (hsCRP), and kidney function (eGFR). Additional important patient and disease characteristics included sex, preceding emotional trigger, and dyslipidaemia. Based on bootstrap selection frequency and clinical considerations, we included five variables in the BioTAK score: NT-proBNP, PAM, sLOX-1, LDL-C, and sex.

**Figure 1:**
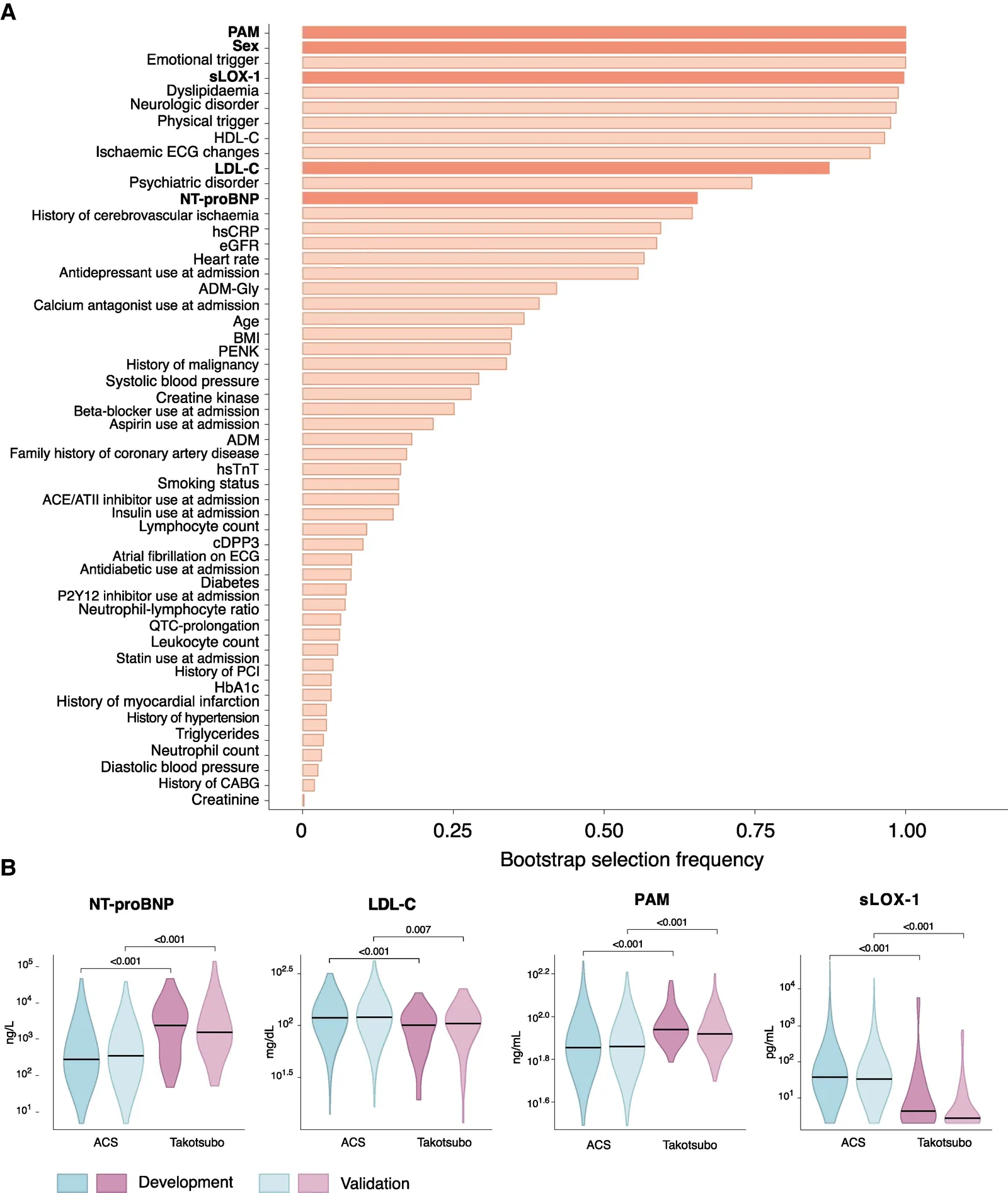
Variable selection for differentiating takotsubo syndrome from acute coronary syndrome. (*A*) Variable importance of predictor variables available before coronary angiography in patients with ACS or TTS in the development cohort. Bars represent the bootstrap selection frequency across 1000 bootstrap resamples, each analysed using elastic-net regression. Higher values indicate that a variable is more reliably associated with takotsubo syndrome. (*B*) Violin plots of selected biomarkers levels in patients with takotsubo syndrome and acute coronary syndrome in the development and validation cohorts. Horizontal lines indicate medians. Groups were compared using the Mann–Whitney *U* test. ACE, angiotensin-converting enzyme; ACS, acute coronary syndrome; ADM, adrenomedullin; ADM-Gly, glycine-extended adrenomedullin; ATII, angiotensin II; BMI, body mass index; CABG, coronary artery bypass grafting; cDPP3, circulating dipeptidyl peptidase 3; CK, creatine kinase; ECG, electrocardiogram; eGFR, estimated glomerular filtration rate; HbA1c, glycated haemoglobin; HDL-C, high-density lipoprotein cholesterol; hsCRP, high-sensitivity C-reactive protein; hsTnT, high-sensitivity troponin T; LDL-C, low-density lipoprotein cholesterol; N/L, neutrophil/lymphocyte; NT-proBNP, N-terminal pro-B-type natriuretic peptide; PAM, peptidylglycine α-amidating monooxygenase; PCI, percutaneous coronary intervention; PENK, proenkephalin; sLOX-1, soluble lectin-like oxidized low-density lipoprotein receptor-1

### Performance of the BioTAK score

BioTAK showed excellent discrimination between TTS and ACS in the development cohort (AUC .97, 95% CI .95–.98), showing consistent results on internal bootstrap validation (see Supplementary data online, *Table S5*). Similar performance was observed in the validation cohort (AUC .93, 95% CI .90–.96) (*Figure 2*). Calibration was acceptable in both cohorts (development cohort: calibration-in-the-large .27, 95% CI −.17–.70, *P* = .228, calibration slope 1.18, 95% CI .98–1.39, *P* = .084; validation cohort: calibration-in-the-large −.07, 95% CI −.44–.30, *P* = .726, calibration slope 1.01, 95% CI .85–1.18, *P* = .874; *Figure 2* and Supplementary data online, *Figures S3* and *S4*). Decision curve analyses suggested favourable clinical utility (see Supplementary data online, *Figure S5*). The non-subjective, five-item BioTAK score was non-inferior to the established InterTAK diagnostic score,^5^ which requires seven clinical variables including psychiatric and neurological history (delta AUC .00, 95% CI −.01–.02, *P* < .001), to the score by Dagrenat *et al*.,^56^ which requires six variables including psychiatric history and cardiac imaging (delta AUC .04, 95% CI .02–.06, *P* < .001) and to established biomarker ratios NT-proBNP/hsTnT (delta AUC .22, 95% CI .16–.28, *P* < .001), hsTnT/CK (delta AUC .46, 95% CI .38–.54, *P* < .001) and NT-proBNP/CK (delta AUC .21, 95% CI .15–.27, *P* < .001). BioTAK provided consistent performance across clinical subgroups (see Supplementary data online, *Table S4*) and maintained robust performance on complete case analysis (see Supplementary data online, *Tables S6* and *S8*).

**Figure 2:**
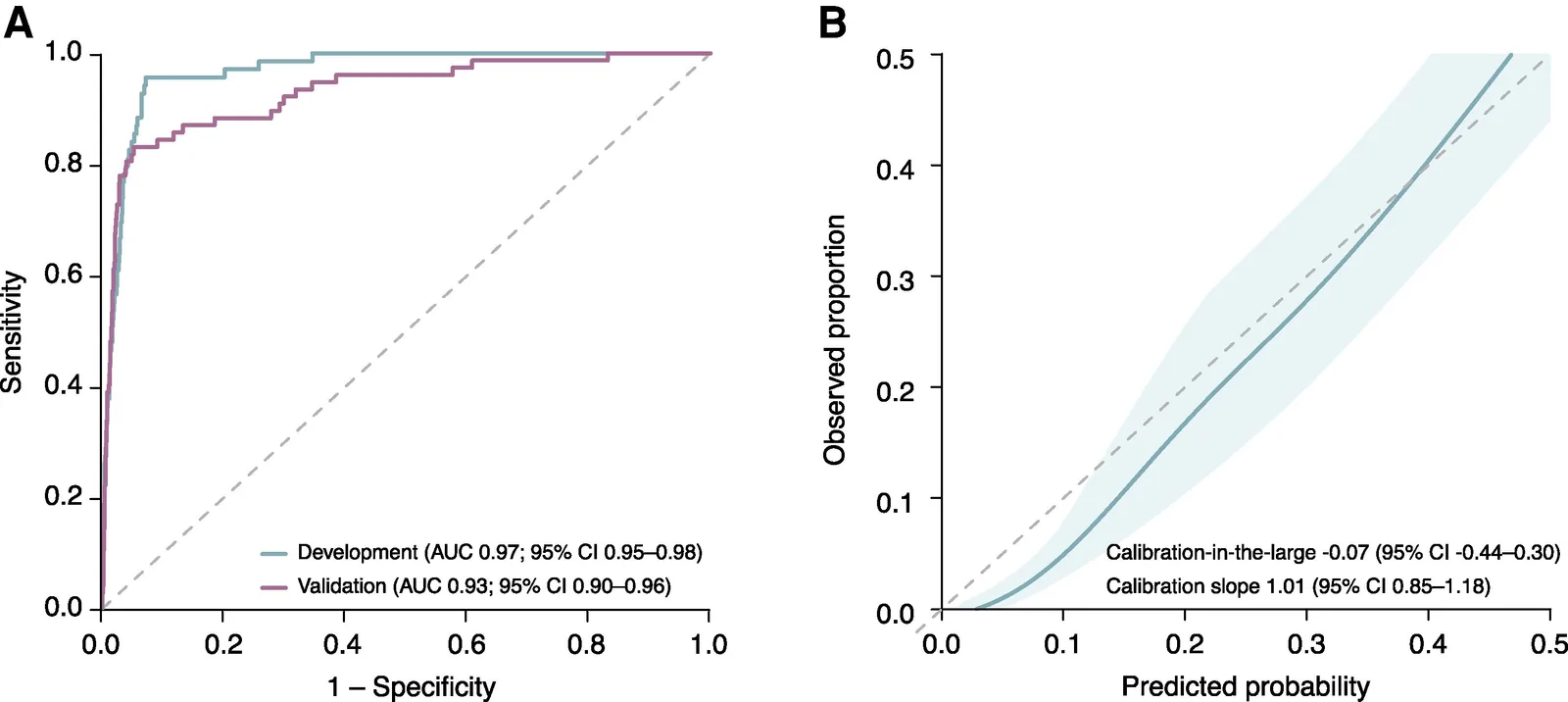
Performance of the BioTAK score. (*A*) Receiver operating characteristic curves for differentiating takotsubo syndrome from acute coronary syndrome in the development and validation cohorts. (*B*) Calibration plot of the BioTAK score in the validation cohort. The smoothed calibration curve was constructed using a restricted cubic spline function with 5 knots. In panel B, the shaded area represents the 95% CI. The dashed line indicates perfect calibration. The solid line represents the smoothed calibration curve. AUC, area under the receiver operating characteristic curve; CI, confidence interval

### Patient stratification according to BioTAK

In the development cohort, the BioTAK score classified 1583 (86.8%), 196 (10.8%), and 44 (2.4%) patients as having low (<27 points), intermediate (27–32 points), and high (≥33 points) probability of TTS, respectively (*Figure 3* and Supplementary data online, *Figure S7*). Thus, 1627 of 1823 patients (89.2%) were assigned to either the low- or the high-probability group. Score values at or above the high-probability threshold, had a specificity of 99.0% (95% CI 98.4–99.3) and a PPV of 59.1% (95% CI 44.4–72.3) to identify TTS. Values above the very-high-probability threshold (≥36 points) had a specificity of 99.9% (99.7–100) and a PPV of 88.9% (56.5–98.0). For ruling out TTS, score values below the low-probability threshold, had a sensitivity of 95.7% (95% CI 88.0–98.5) and a NPV of 99.8% (95% CI 99.4–99.9). Values below the very-low-probability threshold (≤21 points) had a sensitivity of 98.6% (92.2–99.7) and a NPV of 99.9% (99.5–100). Consistent results were obtained in patients presenting with and without ST-segment elevation (see Supplementary data online, *Figures S8* and *S9*), across other clinical subgroups, and on external validation (see Supplementary data online, *Table S9*). When assuming a different prevalence of TTS in the intended use population, BioTAK provided robust PPV and NPV across the entire range of plausible prevalence values (see Supplementary data online, *Table S10*) suggesting that the score could aid rule-in and rule-out TTS in different clinical settings.

**Figure 3:**
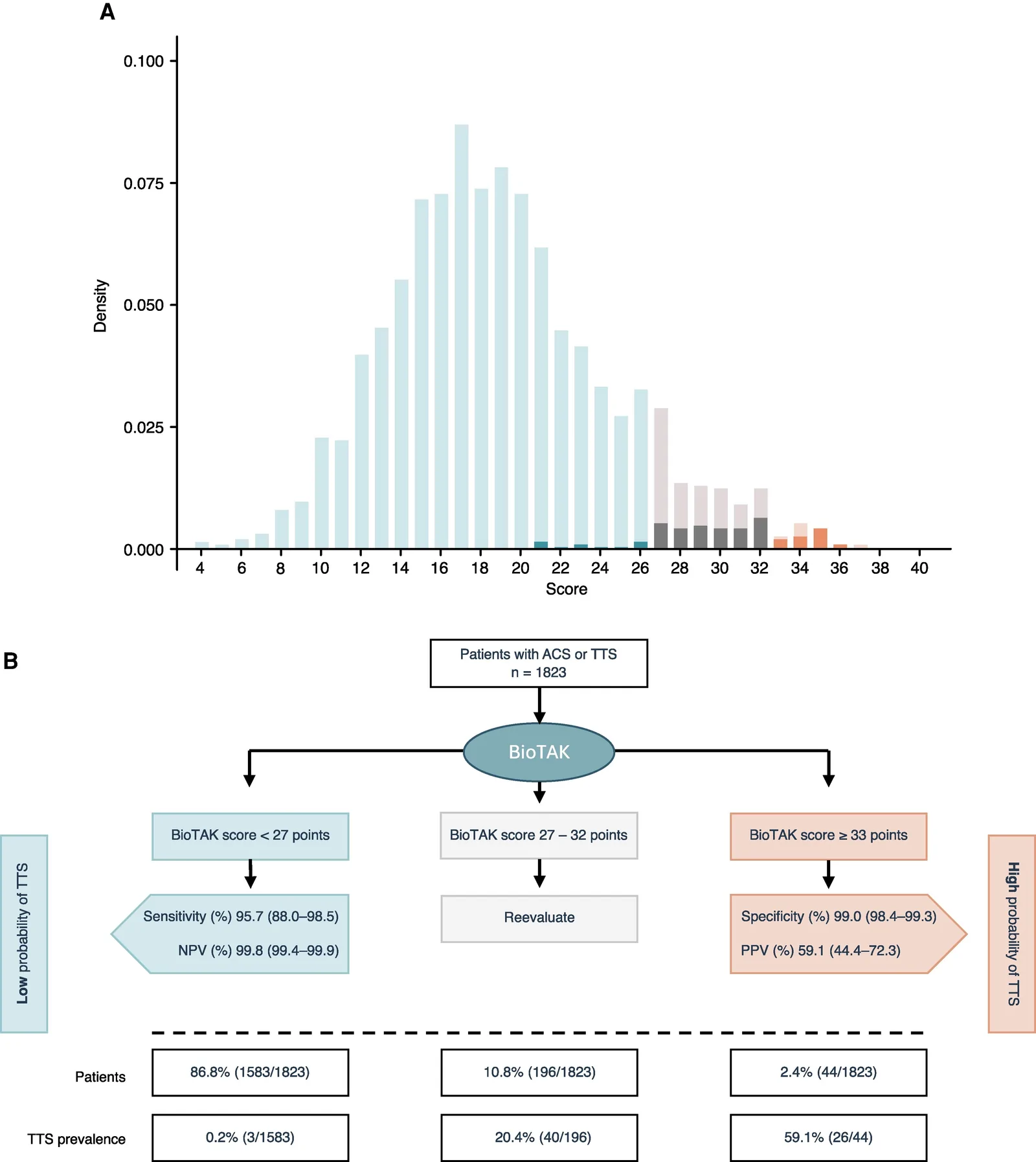
Patient stratification according to BioTAK. (*A*) Distribution of score values stratified by diagnostic outcome and (*B*) patient stratification using the BioTAK diagnostic score and its rule-in and rule-out thresholds. Darker bar segments indicate patients with a final diagnosis of takotsubo syndrome. Colours indicate the pre-specified low-probability (<27 points), intermediate-probability (27–32 points) and high-probability (≥33 points) categories. Results refer to the development cohort, in which patients were enrolled in the same period and study centre. NPV, negative predictive value; PPV, positive predictive value

## Discussion

Leveraging biobanks of overlapping prospective patient cohorts, we have developed and validated a novel, biomarker-based score for early differentiation of TTS from ACS (Structured Graphical Abstract). To our knowledge, this is the first non-subjective tool for early detection of TTS before ICA.

TTS has remained a diagnostic challenge, largely due to its overlap with ACS in terms of clinical presentation. Non-invasive diagnostic tools are warranted, not only to reduce the need for diagnostic ICA and procedure-related complications, but also to conserve healthcare resources. Diagnostic ICA should be minimized in patients with disproportionate risks,^62^ particularly in the presence of comorbidities that are frequently associated with TTS, such as intracranial bleeding, sepsis, hyperthyroidism, acute exacerbation of chronic obstructive pulmonary disease, and anxiety disorders.

Previous diagnostic scores depended on the availability of detailed neurological and psychiatric information and on cardiac imaging.^5,56^ However, reliable history taking in emergency settings is often compromised by haemodynamic instability, language barriers, altered mental status, or respiratory distress. Several small studies have suggested traditional cardiac biomarkers and biomarker ratios for differentiating TTS from ACS.^56,63–65^ However, validation in a large international cohort showed limited diagnostic accuracy.^6^ Another pilot study suggested promising diagnostic properties of miRNAs,^7^ yet validation of these results is pending and miRNA testing has remained barely used in clinical practice due to its high costs, long turnaround time, and limited accessibility. These limitations highlight the need for diagnostic strategies that combine biological rationale with clinical applicability.

Protein and peptide biomarkers meet many of these requirements, owing to their biochemical stability and the comparably lower costs of immunoassay-based testing in clinical laboratories and the increasing availability of point-of-care tests. Therefore, we sought to investigate a panel of measurable protein and peptide biomarkers covering several pathophysiological domains.

The newly developed simple five-item BioTAK score uses biomarkers that can be measured with validated commercially available assays without requiring neuropsychiatric information or cardiac imaging. BioTAK incorporates biomarkers of myocardial stress (NT-proBNP), plaque instability (sLOX-1), sympathetic activation and anxiety modulation (PAM), and lipid metabolism (LDL-C).

sLOX-1 is the soluble form of an endothelial scavenger receptor, highly expressed in the shoulder region of atherosclerotic plaques,^66^ with circulating levels mirroring endothelial activation and plaque instability.^67^ In previous studies, sLOX-1 has proven useful for distinguishing ACS from chronic coronary syndrome or healthy controls.^68,69^ Circulating sLOX-1 levels were lower in TTS compared with ACS, possibly reflecting the absence of acute plaque rupture and erosion as the primary pathophysiological mechanism of TTS.^70^

PAM is an enzyme present in blood and cerebrospinal fluid that catalyses the C-terminal amidation of peptide hormones and thereby activates numerous bioactive peptides involved in sympathetic vasoconstriction and anxiety regulation in the limbic system including in the hippocampus and the amygdala.^40,41^ Circulating PAM levels were higher in TTS compared with ACS, potentially reflecting the pronounced sympathetic activation, microvascular dysfunction, and emotional alteration that characterize TTS.^70^

NT-proBNP is released upon ventricular wall stress indicating elevation of left ventricular end-diastolic pressure observed in TTS, in line with its clinical classification as an acute heart failure syndrome.^1^ LDL-C is a main driver of atherosclerotic plaque formation, tightly linked to ACS but not implicated in TTS.

Integrating these biomarkers, the BioTAK score demonstrated excellent discrimination, good calibration, and potential clinical utility on internal and external validation. Applying predefined diagnostic thresholds, BioTAK provided excellent specificity and a moderate PPV for ruling in TTS while maintaining an excellent NPV and high-sensitivity for ruling out TTS. BioTAK supports standardized identification of TTS early in the clinical care pathway, stratifying almost 90% of patients towards a low- or high-probability group.

Given that the presence of ST-segment elevation or of very-high-risk characteristics mandate emergent invasive treatment,^71^ considerations regarding the indication for ICA primarily involve ACS patients without these criteria. Indeed, the requirement for and the optimal timing of ICA in ACS without ST-segment elevation and without high-risk criteria has remained uncertain.^72–74^ Therefore, BioTAK can support timely diagnostic evaluation for TTS and might help identify patients in whom ICA can safely be deferred. The score may have its greatest practical utility in patients with non-ST-elevation (NSTE)-ACS without very high-risk criteria, such as haemodynamically stable elderly patients at low-to-intermediate mortality risk. Notably, postmenopausal women, who represent the primary population affected by TTS, most commonly present with ACS without ST-segment elevation. Within this framework, BioTAK may thus support patient triage and inform personalized decisions regarding both the indication for and the timing of invasive procedures.

Standardized early diagnosis of TTS may shape the design of future trials. The BioTAK score could facilitate the recruitment or exclusion of patients with TTS at scale, thereby supporting research on novel treatment strategies addressing disease-specific mechanisms.

### Strength and limitations

Our study has several strengths. First, the prospective collection of biospecimens within a structured biobanking framework, combined with predefined inclusion and exclusion criteria forms a robust basis for this study. Harnessing overlapping patient cohorts, we developed the BioTAK score based on consecutively enrolled patients from the same study centres, during the same observation period to minimize potential bias. Blood samples were processed, stored, and analysed using standardized protocols reducing the risk of preanalytical or analytical bias and strengthening the validity of the findings. Second, to the best of our knowledge, this study represents the largest biomarker study on TTS to date. Third, BioTAK represents the first non-subjective tool for early identification of TTS.

Several limitations should be acknowledged. First, although external validation was performed, both cohorts are based on closely related registry infrastructures imposing structural constraints on the study design, and further validation in additional cohorts is desirable. Second, in patients with ST-segment elevation or high-risk criteria, ICA may be mandated despite a high probability of TTS. However, early identification of TTS in these settings may facilitate future clinical and translational research. Third, routine hospital laboratory tests for measuring sLOX-1 and PAM are not available at present, which may limit immediate clinical applicability. However, both assays demonstrate several characteristics that suggest high translational potential for automated clinical immunoassay platforms, including a simple immunoassay format, low sample volume requirements, excellent analytical precision, high analyte stability, and a broad operational temperature range. Next, turnaround times of current assays for sLOX-1 and PAM are ∼3–5 h. Yet, they are well below the guideline-recommended time window for early invasive management in patients with suspected NSTE-ACS. In addition, even shorter turnaround times are expected after assay optimization and transfer to automated platforms. Moreover, we acknowledge that the wide CIs of diagnostic accuracy metrics in several patient subgroups may potentially not only be due to the small number of patients and TTS diagnoses in these analyses but could also hint towards model misspecification. Further, positive and negative predictive values are inherently dependent on the disease prevalence and may thus vary across clinical settings and populations (see Supplementary data online, *Table S10*). Finally, while the BioTAK score was designed to facilitate early diagnosis, its impact on routine clinical care workflows and patient outcomes is unknown, requiring further investigation in implementation studies.

## Conclusions

TTS is characterized by a disease-specific biomarker profile that reflects pathophysiological differences from ACS and can support early detection. The biomarker-enhanced BioTAK score may facilitate accurate early rule-in and rule-out of TTS before ICA. BioTAK has the potential to streamline diagnostic pathways and reduce invasive diagnostic procedures in selected patients presenting with suspected ACS.

## Supplementary Material

ehag597_Supplementary_Data

## Declarations

### Data Availability

The data underlying this article cannot be shared publicly due to data protection regulations related to the different patient cohorts involved in this study. Requests for the data and additional documents related to this study should be made to the corresponding authors.

### Pre-registered Clinical Trial Number

The identifiers are ClinicalTrials.gov Identifier NCT01000701 (SPUM-ACS) and ClinicalTrials.gov Identifier NCT01947621 (InterTAK).
