# Supplementary material for: Takotsubo syndrome diagnosis: the biomarker-based BioTAK score
Source: Eur Heart J. 2026 Aug 31;47(41):6166–79. doi: 10.1093/eurheartj/ehag597 (PMC13739320; doi:10.1093/eurheartj/ehag597)
Supplement: Supplementary file 1 [file ehag597_supplementary_data.pdf]

-- ONLINE SUPPLEMENTARY APPENDIX --

Takotsubo syndrome diagnosis: the biomarker-based BioTAK score

Florian A. Wenzl,[1,2,3,4†*] Victor K. Schweiger,[5†*] Maria A. Smolle,[6] Daniel Zech,[1] Alexander Akhmedov,[1] Peizhi Wang,[1,7,8] Tetiana Lapikova-Bryhinska,[1] Yujing Wang,[1] Luca Liberale,[9,10] Karine Santos,[11] Yulia Ilina,[12] Simon Kraler,[1,13] Andreas Bergmann,[12] Paul Kaufmann,[12] Florian Kahles,[14] Arnold von Eckardstein,[15] Marco Roffi,[16] Lorenz Räber,[13] Victoria L. Cammann,[5] Jelena R. Ghadri,[1,17,18] Davide Di Vece,[17] Thomas F. Lüscher,[1,19,20,21*] and Christian Templin,[1,17,18*] on behalf of the InterTAK and SPUM-ACS investigators

1 Center for Molecular Cardiology, University of Zurich, Wagistrasse 12, 8952 Schlieren, Switzerland.
2 National Disease Registration Service, NHS England, 10 South Colonnade, Canary Wharf, London, UK.
3 Department of Cardiovascular Sciences, University of Leicester, University Road, Leicester, LE1 7RH, UK.
4 Division of Cardiovascular Medicine, Radcliffe Department of Medicine, University of Oxford, John Radcliffe Hospital, Headington, Oxford OX3 9DU, UK.
5 Department of Cardiology, University Hospital Zurich and University of Zurich, Rämistrasse 100, 8091 Zurich Switzerland.
6 Comprehensive Cancer Center Graz, Medical University of Graz, Auenbruggerplatz 19, 8030 Graz, Austria.
7 Department of Cardiology, State Key Laboratory of Complex Severe and Rare Diseases, Peking Union Medical College Hospital, Beijing, China.
8 Department of Internal Medicine, State Key Laboratory of Complex Severe and Rare Diseases, Peking Union Medical College Hospital, Beijing, China.
9 First Clinic of Internal Medicine, Department of Internal Medicine, University of Genoa, Viale Benedetto XV 6, 16132 Genoa, Italy.
10 AOM-IRCCS Ospedale Policlinico San Martino, Largo Rosanna benzi 19, 16132 Genoa, Italy.
11 4TEEN4 Pharmaceuticals GmbH, Neuendorferstraße 15B, 16761 Hennigsdorf, Germany.
12 PAM Theragnostics GmbH, Neuendorfstraße 15B, 16761 Hennigsdorf, Germany.
13 Department of Cardiology, Bern University Hospital, University of Bern, Freiburgstrasse 18, 3010 Bern, Switzerland.
14 Department of Internal Medicine I, University Hospital Aachen, RWTH Aachen, Pauwelsstraße 30, 52074 Aachen, Germany.
15 Institute of Clinical Chemistry, University Hospital Zurich, Rämistrasse 100, 8091 Zurich, Switzerland.
16 Service de Cardiologie, Hôpitaux Universitaires de Genève, Rue Gabrielle-Perret-Gentil 4, 1205 Geneva, Switzerland.
17 Department of Internal Medicine B, University Medicine Greifswald, Ferdinand-Sauerbruch-Straße, 17475 Greifswald, Germany.
18 SwissCardioVascular Clinic (SwissCVC), Private Hospital Bethanien, Toblerstrasse 51, 8044 Zurich, Switzerland.
19 Heart Division, Royal Brompton and Harefield Hospitals, GSST, Syndey Street, London SW3 6NP, UK.
20 School of Cardiovasulcar and Metabolic Medicine, King's College, Strand, London, WCR2 2LS, UK.
21 National Heart and Lung Institute, Imperial College, Guy Scadding Building, Cale Street, London SW3 6LY, UK.

† Contributed equally as first authors
* Jointly directed the study

Running Title: Biomarkers for early diagnosis of takotsubo syndrome

Correspondence

Professor Thomas F. Lüscher, MD, FRCP, FESC
Director of Research, Education & Development
Royal Brompton & Harefield Hospital
Sydney Street
London SW3 6NP, UK
Email: cardio@tomluescher.ch
Phone: +41 44 250 40 97

Dr. Florian A. Wenzl, MD, PhD, FESC
National Disease Registration and Analysis Service
National Health Service
10 South Colonnade, Canary Wharf
London E14 4PU, UK
E-mail: florian.wenzl1@nhs.net
Phone: +41 76 749 42 50

Table of Contents

Description of the statistical analyses (detailed)......3
Table S1: Rationale for biomarker measurement 6
Table S2: Measurement of emerging biomarkers......8
Table S3: Sample size considerations......9
Table S4: Model performance across clinical subgroups......10
Table S5: Internal bootstrap validation of model performance......11
Table S6: Model performance on complete case analysis 12
Table S7: Missing data summary 13
Table S8: Completeness of BioTAK scocre variables......15
Table S9: Diagnostic accuracy across clinical subgroups 16
Table S10: Diagnostic accuracy across different prevalence values 18
Figure S1: Flow-chart of the study......19
Figure S2: Violin plots of biomarker levels in development and validation cohorts......20
Figure S3: Calibration plot in development cohort 22
Figure S4: Bias-corrected calibration curves 23
Figure S5: Decision curves of the BioTAK score in development and validation cohorts......24
Figure S6: Nomogram for calculation of the BioTAK score......25
Figure S7: Stratification of patients with possible takotsubo syndrome in the validation cohort 26
Figure S8: Stratification of patients with possible takotsubo syndrome presenting with ST-segment elevation 27
Figure S9: Stratification of patients with possible takotsubo syndrome presenting without ST-segment elevation 29
Figure S10: STARD Checklist 31
Figure S11: TRIPOD Checklist 33
References 34

Description of the statistical analyses (detailed)

Patient cohorts

We combined two temporally and geographically overlapping acute coronary syndrome (ACS) and takotsubo syndrome (TTS) registries with biobanks into a large diagnostic cohort of consecutive patients with possible TTS at the same point in the clinical care pathway. A biomarker-enhanced diagnostic score was developed in consecutive patients treated at University Hospital Zurich, Switzerland between 07/2011 and 01/2018 and externally validated in patients presenting at a different time and/or at a different study centre (***Figure S1***).

The SPUM-ACS Biomarker registry (ClinicalTrials.gov Identifier: NCT01000701), an investigator-initiated, prospective, multicentre cohort study included patients presenting with acute coronary syndrome at participating Swiss university hospitals. SPUM-ACS is characterised by its rigorous methodological framework designed to generate high-quality, real-world evidence on extensive phenotypic profiling, a comprehensive biobank, and clinical follow-up.[1] Patients with a main diagnosis of ACS were included if they were aged 18 years or older and were admitted within 5 days of symptom onset.[1] Individuals with severe physical disability, inability to provide informed consent, or non-cardiac life expectancy < 1 year were excluded.[2] Diagnoses of ACS were independently validated by investigators at local study centres.[1,2] Treating physicians were advised to apply guideline-directed management.[1,2]

The InterTAK registry (ClinicalTrials.gov Identifier: NCT01947621; www.takotsubo-registry.com) is an investigator-initiated prospective observational study aimed for better insight into the clinical profile and pathophysiology of TTS. Data on demographical characteristics, clinical phenotype, laboratory values, electrocardiography parameters, echocardiography parameters, coronary angiography parameters, medication and outcome are systematically collected. EDTA blood samples were obtained at the time of presentation before coronary angiography, as described previously.[1,2]

In both registries, ethylenediaminetetraacetic acid (EDTA) blood samples were obtained at the time of presentation before any intervention.[1,2]

Calculation of established diagnostic scores and biomarker ratios

The InterTAK diagnostic score was calculated according to Ghadri *et al*.[3] using the following variables: sex (binary), emotional trigger (binary), physical trigger (binary), absence of ST-segment depression (binary), psychiatric disorders (binary), neurological disorders (binary), QTc prolongation (binary). To translate integer score values into probabilities, the association between the score value and TTS was quantified with logistic regression analysis. Calculation of the score by Dagrenat et al.[4] was based on sex (binary), age (continuous), natriuretic peptide level (continuous), troponin level (continuous), left ventricular ejection fraction [LVEF] (continuous), and history of psychiatric disease (binary). Given that our dataset provides high-sensitivity Troponin T [hsTnT] and N-terminal pro B-type natriuretic peptide [NT-proBNP] instead of the originally used TnI and BNP, the score was recalibrated by re-estimating all model coefficients using logistic regression. Further, the established biomarker ratios NT-proBNP/hsTnT ratio, hsTnT/creatine kinase [CK] and NT-proBNP/CK were calculated.

Variable selection and score development

The selection of candidate variables was based on the literature as well as clinical considerations, such as their availability before invasive coronary angiography, and included all predictor variables shown in ***Figure 1***. Variable selection and model development was performed on data from the development cohort. Biomarkers were log-transformed to reduce skewness, as reported previously.[5-10] We evaluated (non-parametric) bootstrap selection frequency using elastic-net regression for identifying the most reliable predictors of a TTS diagnosis. We generated 1000 bootstrap resamples of the original dataset and fitted an elastic-net logistic regression model to each resample using the *glmnet* package. We set the mixing parameter alpha to 0.5 and the measure of the loss function to AUC. The regularisation parameter lambda was optimized (i.e., tuned) within each bootstrap sample using k-fold (10-fold) cross-validation, applying the one-standard-error rule (lambda.1se). Predictor variables with nonzero coefficients at the optimized lambda value were recorded as selected. Selection frequencies were calculated as the proportion of bootstrap samples in which a specific predictor variable was selected; higher values indicate that a variable is more reliably associated with takotsubo syndrome. Based on both data-driven variable importance (bootstrap selection frequency) and clinical considerations, a final set of five non-subjective early predictors (i.e., NT-proBNP, PAM, sLOX-1, LDL-C, and sex) were selected for score development.

The selected predictors were incorporated in a logistic regression model for predicting TTS. To reduce the influence of extreme values, we truncated continuous predictors at the first and 99th percentile, as reported

previously.[5,11,12] The linearity assumption was evaluated using restricted cubic splines with four knots placed at the default recommended percentiles (5th, 35th, 65th, and 95th)[13] within a multivariable logistic regression model. Non-linearity was assessed using the Wald χ^2 test for the non-linear spline components using the analysis of variance (ANOVA) decomposition of the spline terms. Only log-transformed PAM demonstrated statistically significant evidence of non-linearity. To account for this, we modelled log-transformed PAM using a restricted cubic spline function with four knots placed at the above-described default positions, as reported previously.[6,14] To prevent model overfitting given the limited size of the development dataset, we applied an L2 (Ridge) penalty. Finally, the model was transformed into a nomogram-based score yielding integer score points.

Internal validation using bias (optimism)-correction

Internal validation of model performance was performed by calculating bias (optimism)-corrected performance metrics using non-parametric bootstrap resampling. Within the framework of the *validate()* and *calibrate()* functions of Frank Harrell's regression modelling strategies (*rms*) package applying 1000 bootstrap replicates (***Table S5***). A smoothed bias (optimism)-corrected calibration curve was constructed using locally weighted scatterplot smoothing (lowess) argument of the *calibrate()* function with default parameters (***Figure S4***).

Evaluation of score performance and diagnostic accuracy

We assessed various aspects of model performance. Discriminatory performance was evaluated by the area under the receiver operating characteristic curve (AUC). The AUC represents the probability that the model, if given a randomly chosen patient with TTS and a randomly chosen patient with ACS, will assign a higher score to the patient with TTS. The ideal value of the AUC is 1. We derived 95% CIs for the AUC estimates using the DeLong method.[15,16] Calibration was evaluated by constructing smoothed calibration curves using the *val.prob.ci.2()* function of the *CalibrationCurves* package by setting the smooth argument to "rcs" using default parameters, which generates calibration curves based on restricted cubic splines with five knots. In addition, calibration was quantified by calculating the calibration-in-the-large and the calibration slope. Calibration-in-the-large measures whether a model over- or underpredicts a risk systematically and is quantified as the intercept of the calibration plot with an optimal value is 0. The calibration slope quantifies the spread of predicted risks, with an ideal value of 1. For calibration in the large and calibration slope, 95% CIs were estimated using the Wald method. Overlap of the smoothed calibration curve with the diagonal indicates ideal calibration. Clinical utility was evaluated using decision curve analyses. This approach implicitly accounts for both discrimination and calibration and extends the model evaluation to consider the ramifications for clinical decision making. The accuracy of the BioTAK score using its diagnostic probability thresholds was assessed by calculating the sensitivity, specificity, positive predictive value (PPV), and negative predictive value (NPV). Sensitivity was calculated as TP / (TP + FN). Specificity was calculated as TN / (TN+FP). NPV was calculated as TN / (TN+FN). PPV was calculated as TP / (TP + FP). Optimal value for each of the four metrics is 100%. For sensitivity, specificity, NPV and PPV, CIs were obtained with the Wilson method.[17] TP denotes number of true positive results, TN number of true negative results, FP number of false positive results, and FN number of false negative results.

Comparison of model performance

We compared the performance of the newly developed BioTAK score with the established InterTAK diagnostic score[3], the diagnostic score proposed by Dagrenat et al.[4] and with established biomarker ratios (NT-proBNP/hsTnT ratio, hsTnT/creatine kinase [CK] ratio, NT-proBNP/CK ratio) within a non-inferiority framework. We conducted a one-sided hypothesis test using a non-inferiority margin of -0.05 delta AUC (positive values indicate better performance of BioTAK). We chose this value as non-inferiority margin considering that 1) differences in AUC larger than of 0.05 may be perceived as notably lower performance of the novel non-subjective BioTAK score compared to the established diagnostic scores and biomarker ratios, thus limiting the clinical adoption, 2) it aligns with the default setting in established statistical software packages (e.g., Analyse-it®), and 3) this margin has been reported previously for comparing prediction models.[7]

Multiple imputation

Completeness, representation, and plausibility of the data were checked as appropriate.[18] Data were multiply imputed using m = 20 imputed datasets with 10 iterations,[5] as reported previously.[5,19-21] We employed random forest models for continuous, ordinal, and binary variables to impute missing data under the missing at random assumption. The imputation models contained all predictor variables, the diagnostic outcome indicator, and the date of cohort entry. We chose to use random forest imputation models due to high computational efficiency and their ability to handle complex non-linear relationships between imputed variables, as reported previously.[16,19]

Convergence was assessed visually. Imputed data were visualised and compared to observed data using strip plots. Development and validation cohorts were imputed separately, as reported previously.[7,15,16,22] Variable selection was based on a single imputed dataset.[20,21,23] During development of the prediction model, coefficients of the predictor variables were derived from each imputed data set. Subsequently, the coefficients were pooled across the 20 datasets using Rubin's Rules[24,25] to ultimately obtain one prediction model.[5,26] Analyses on model accuracy were carried out on a single imputed dataset.[20,21,27-31] To assess the potential impact of imputation on the results, sensitivity analyses were conducted using complete cases (***Table S8***).

Software packages

Analyses were performed R (version 4.2.2). The software environment in R for the analyses was created by the R packages: *dplyr, caret, mice, pROC, rms, epiR, pracma, nomogramFormula, stats, CalibrationCurves, rmda, tableone, ggplot2, vip, mcradds, glmnet, Hmisc, car*

Table S1: Rationale for biomarker measurement

	Type of biomarker	Rationale	References
**Myocardial wall stress and haemodynamic disturbance**			
NT-proBNP	Established	Released into the blood stream as result of myocardial wall stress; substantially increased in patients with TTS	32-34
ADM	Emerging	Peptide synthesised by endothelial and vascular smooth muscle cells; leads to vasodilatation; increased levels in patients with heart failure	35-37
ADM-Gly	Emerging	Inactive glycine-extended precursor of ADM, elevated in patients with heart failure	38-40
cDPP3	Emerging	Aminopeptidase that is released into circulation upon cell death and degrades different bioactive peptides including angiotensin II; modulates systemic inflammatory pathways, disturbs blood pressure regulation and regulates immune response	1,41-43
**Inflammation**			
hsCRP	Established	Acute-phase protein indicating systemic inflammation; increased hsCRP levels associate with lower LVEF, prolonged in-hospital stay and recurrence of disease in patients with TTS	44,45
Leukocytes	Established	Constitutes marker of systemic inflammation and stress; comparable levels in patients with ACS and TTS	3,46
Neutrophil-lymphocyte-ratio	Established	Elevated N/L-ratio associated with in-hospital complications and poor survival in patients with TTS	47
Neutrophils	Established	Comparable levels between patients with ACS and TTS; higher neutrophil levels in TTS patients with physical trigger compared to patients with emotional or no identifiable trigger	47
Lymphocytes	Established	Comparable levels between patients with ACS and TTS	47
**Lipid and glucose metabolism**			
LDL-C	Established	Causally involved in development of atherosclerotic cardiovascular disease	48
HDL-C	Established	High levels associated with lower risk of coronary artery disease	49
Triglycerides	Established	Increased triglyceride levels associate with development of coronary artery disease, myocardial infarction, ischaemic heart disease and overall mortality	50,51
HbA1c	Established	High levels indicate poor glycaemic control and are associated with increased risk for cardiovascular disease and overall mortality	52
**Myocardial injury**			
hsTnT	Established	TnT released into blood stream upon myocardial injury; TnT levels at admission comparable between patients with TTS and ACS; peak TnT levels lower in TTS than ACS	34,53
CK	Established	Released into plasma upon myocardial injury; only slightly elevated in patients with TTS	34,54,55
**Endothelial activation and plaque disruption**			
sLOX1	Emerging	Soluble form of endothelial scavenger receptor, highly expressed in the shoulder region of atherosclerotic plaques, circulating levels mirroring endothelial activation and plaque instability	2,56-59

**Vasoconstriction and anxiety regulation**			
PAM	Emerging	Enzyme in blood and cerebrospinal fluid essential for amidation of peptide hormones, involved in sympathetic vasoconstriction and anxiety regulation in the limbic system including in the hippocampus and the amygdala	38,60-62
**Kidney function**			
eGFR	Established	Estimated glomerular filtration based on serum creatinine and demographic variables; eGFR is reduced in a substantial proportion of patients presenting with ACS; low eGFR associated with higher in-hospital mortality	63
Creatinine	Established	Cleavage product of creatine phosphate from muscle metabolism, high levels indicate impaired glomerular filtration; associated with adverse outcomes	15,64
PENK	Emerging	Stable endogenous polypeptide reflecting glomerular dysfunction and tubular damage; serves as a real-time biomarker of acute kidney injury	7,65-67

ACS, acute coronary syndrome; ADM, adrenomedullin; ADM-Gly, glycine-extended ADM; CK, creatine kinase; cDPP3, circulating dipeptidyl peptidase 3; eGFR, estimated glomerular filtration rate; HbA1c, haemoglobin A1c; HDL-C, high-density lipoprotein cholesterol; hsCRP, high-sensitivity C-reactive protein; hsTnT, high-sensitivity troponin T; LDL-C, low-density lipoprotein cholesterol; N/L, neutrophil/lymphocyte; NT-proBNP, N-terminal pro B-type natriuretic peptide; PAM, peptidylglycine α-amidating monooxygenase; PENK, proenkephalin; sLOX-1, soluble lectin-like oxidised low-density lipoprotein receptor-1; TTS, takotsubo syndrome

Table S2: Measurement of emerging biomarkers

Marker	Assay	Manufacturer	Sample type	Intra- and inter-assay coefficients of variation	References
sLOX-1	Enzyme-linked immunosorbent assay	Thermo Scientific™ Pierce™, Waltham, MA, USA	EDTA plasma	Intra-assay coefficient of variation 3.36%<br>Inter-assay coefficient of variation 5.14%	68
cDPP3	Immunoluminometric assay	4TEEN4 Pharmaceuticals GmbH, Hennigsdorf-Berlin, Germany	EDTA plasma	Intra-assay coefficient of variation 3.8%<br>Inter-assay coefficient of variation 5.2%	1
PENK	Sandwich immunoassay	Sphingotest penKid, SphingoTec GmbH, Hennigsdorf, Germany	EDTA plasma	Intra-assay coefficient of variation 6.4% (at 50 pmol/L) and 4.0% (at 150 pmol/L)<br>Inter-assay coefficient of variation 9.5% (at 50 pmol/L) and 6.5% (at 150 pmol/L)	7
PAM	Sandwich chemiluminescence immunoassay	PAM Theragnostics GmbH, Hennigsdorf, Germany	EDTA plasma	Intra-assay coefficient of variation 2.2%<br>Inter-assay coefficient of variation 6.7%	69
ADM	Sandwich chemiluminescence immunoassay	SphingoTec GmbH, Hennigsdorf, Germany	EDTA plasma	Intra-assay coefficient of variation 5–10%<br>Inter-assay coefficient of variation 4–8%	70
ADM-Gly	One-step microtiter plate–based chemiluminescence immunometric assay	PAM Theragnostics GmbH, Hennigsdorf, Germany	EDTA plasma	Intra-assay coefficient of variation 4.05%<br>Inter-assay coefficient of variation 7.86%	71

ADM, adrenomedullin; ADM-Gly, glycine-extended ADM; cDPP3, circulating dipeptidyl peptidase 3; EDTA, ethylenediaminetetraacetic acid; PAM, peptidylglycine α-amidating monooxygenase; PENK, proenkephalin; sLOX-1, soluble lectin-like oxidised low-density lipoprotein receptor-1

Table S3: Sample size considerations

	Sample size	Shrinkage	Parameters	C-statistic (AUC)
**Prediction of TTS**				
**Criteria 1**	638	0.900	8	0.90
**Criteria 2**	527	0.882	8	0.90
**Criteria 3**	60	0.900	8	0.90
**Minimum sample size**	638	0.900	8	0.90

This table shows the required sample size for powered model development for the prediction of a binary outcome computed according to the three criteria proposed by Riley *et al.* (2020).[72,73] Given (1) a prevalence of takotsubo syndrome (TTS) of 0.04 in the development cohort, in line with prior studies,[74] (2) an expected c-statistic (AUC) of 0.90, in line with the performance of prior diagnostic models,[3,4] and (3) a total of 8 parameters (5 predictor variables, one of which handled using a 4-knot restricted cubic spline) our calculations suggest that at least 638 patients are required to develop an accurate prediction model for TTS.

AUC, area under the receiver operating characteristic curve

Table S4: Model performance across clinical subgroups

		AUC (95% CI)	Calibration-in-the-large (95% CI)	p-value	Calibration slope (95% CI)	p-value
**Development cohort**						
All patients		0.97 (0.95–0.98)	0.27 (-0.17–0.70)	0.228	1.18 (0.98–1.39)	0.084
Age	≤ 65 years	0.99 (0.99–0.99)	0.65 (-0.27–1.57)	0.166	1.65 (1.11–2.19)	0.019
	> 65 years	0.94 (0.91–0.97)	0.10 (-0.40–0.60)	0.695	1.01 (0.78–1.24)	0.931
Sex	Female	0.89 (0.85–0.93)	0.17 (-0.27–0.61)	0.459	1.06 (0.80–1.31)	0.663
	Male*	0.94 (0.87–1.00)	1.35 (-0.94–3.64)	0.246	1.61 (0.85–2.37)	0.117
ST-elevation	No	0.97 (0.95–0.98)	0.30 (-0.25–0.85)	0.283	1.11 (0.85–1.37)	0.397
	Yes	0.96 (0.94–0.99)	0.23 (-0.47–0.94)	0.511	1.26 (0.92–1.60)	0.136
Diabetes	No	0.97 (0.95–0.98)	0.40 (-0.09–0.89)	0.114	1.19 (0.97–1.41)	0.096
	Yes	0.97 (0.95–0.99)	-0.18 (-1.12–0.76)	0.709	1.23 (0.67–1.79)	0.424
Hypertension	No	0.98 (0.97–0.99)	1.47 (0.44–2.50)	0.005	1.59 (1.11–2.07)	0.015
	Yes	0.96 (0.93–0.98)	-0.08 (-0.57–0.40)	0.735	1.07 (0.83–1.30)	0.578
Dyslipidaemia	No	0.97 (0.96–0.99)	1.34 (0.66–2.01)	<0.001	1.29 (0.99–1.60)	0.060
	Yes	0.95 (0.91–0.99)	-0.88 (-1.53–-0.23)	0.008	1.07 (0.76–1.37)	0.667
Evidence of pre-existing CAD	No	0.97 (0.95–0.98)	0.34 (-0.13–0.80)	0.152	1.17 (0.95–1.38)	0.123
	Yes*	0.98 (0.96–0.99)	-0.16 (-1.39–1.07)	0.801	1.28 (0.60–1.96)	0.412
**Validation cohort**						
All patients		0.93 (0.90–0.96)	-0.07 (-0.44–0.30)	0.726	1.01 (0.85–1.18)	0.874
Age	≤ 65 years	0.94 (0.89–0.99)	0.05 (-0.68–0.77)	0.900	1.02 (0.75–1.30)	0.866
	> 65 years	0.92 (0.87–0.97)	-0.10 (-0.54–0.33)	0.637	1.02 (0.81–1.23)	0.863
Sex	Female	0.89 (0.84–0.94)	-0.02 (-0.41–0.38)	0.935	1.09 (0.84–1.35)	0.469
	Male	0.84 (73.2–94.9)	-0.33 (-1.89–1.24)	0.685	0.92 (0.53–1.30)	0.664
ST-elevation	No	0.94 (0.90–0.98)	0.22 (-0.25–0.69)	0.362	1.04 (0.83–1.25)	0.708
	Yes	0.91 (0.85–0.98)	-0.56 (-1.19–0.07)	0.080	0.94 (0.68–1.21)	0.675
Diabetes	No	0.93 (0.90–0.97)	0.11 (-0.30–0.50)	0.616	1.04 (0.86–1.22)	0.668
	Yes*	0.93 (0.88–1.00)	-1.19 (-2.28–-0.10)	0.032	0.68 (0.41–1.32)	0.555
Hypertension	No	0.91 (0.85–0.97)	0.26 (-0.34–0.87)	0.400	0.93 (0.71–1.14)	0.503
	Yes	0.96 (0.93–0.99)	-0.20 (-0.68–0.28)	0.407	1.22 (0.93–1.50)	0.133
Dyslipidaemia	No	0.91 (0.85–0.96)	0.15 (-0.35–0.65)	0.559	0.90 (0.70–1.10)	0.330
	Yes	0.97 (0.94–0.99)	-0.28 (-0.84–0.28)	0.327	1.19 (0.89–1.49)	0.217
Evidence of pre-existing CAD	No	0.94 (0.91–0.97)	0.36 (-0.06–0.78)	0.097	1.12 (0.92–1.31)	0.231
	Yes*	0.80 (0.61–0.99)	-2.51 (-3.94–-1.09)	<0.001	0.51 (0.08–0.95)	0.023

P-values were calculated using the Wald test. *less than 10 Takotsubo diagnoses in subgroup; evidence of preexisting CAD was defined as recorded prior percutaneous coronary intervention, coronary artery bypass graft or myocardial infarction
AUC, area under the receiver operating characteristic curve; CAD, coronary artery disease; CI, confidence interval

Table S5: Internal bootstrap validation of model performance

	AUC (95% CI)	Calibration-in-the-large (95% CI)	Calibration slope (95% CI)
Development cohort	0.97 (0.95–0.98)	0.30 (-0.14–0.82)	1.18 (0.96–1.39)

AUC, area under the receiver operating characteristic curve; CI, confidence interval

Table S6: Model performance on complete case analysis

	AUC (95% CI)	Calibration-in-the-large (95% CI)	p-value	Calibration slope (95% CI)	p-value
Development cohort	0.95 (0.91–0.98)	0.17 (-0.34–0.78)	0.518	1.11 (0.88–1.33)	0.352
Validation cohort	0.93 (0.88–0.97)	0.32 (-0.18-0.83)	0.209	1.09 (0.87–1.31)	0.422

P-values were calculated using the Wald test. AUC, area under the receiver operating characteristic curve; CI, confidence interval

Table S7: Missing data summary

	Development cohort (n=1823)		Validation cohort (n=1792)	
	Missing	Percentage	Missing	Percentage
Female sex	0	0.0	0	0.0
Age	0	0.0	0	0.0
BMI	44	2.4	40	2.2
Systolic blood pressure	46	2.5	26	1.5
Diastolic blood pressure	51	2.8	25	1.4
Heart rate	27	1.5	20	1.1
ST-segment elevation	0	0.0	0	0.0
ACS type	0	0.0	0	0.0
Takotsubo type	0	0.0	0	0.0
Emotional trigger	277	15.2	..	..
Physical trigger	277	15.2	..	..
Psychiatric disorder	277	15.2	..	..
Neurological disorder	277	15.2	..	..
ECG				
QTc-prolongation	277	15.2	..	..
Atrial fibrillation	277	15.2	..	..
Ischaemia	125	6.9	106	5.9
LVEF	509	27.9	846	47.2
Smoking status	28	1.5	27	1.5
Medical history				
Asthma	277	15.2	..	..
Family history of coronary artery disease	43	2.4	11	0.6
Diabetes	0	0.0	0	0.0
Hypertension	0	0.0	2	0.1
Dyslipidaemia	2	0.1	0	0.0
Prior myocardial infarction	0	0.0	7	0.4
Prior PCI	0	0.0	0	0.0
Prior CABG	0	0.0	0	0.0
Cerebrovascular ischaemia	0	0.0	0	0.0
Malignancy	0	0.0	1	0.1
Medication at admission				
Aspirin	615	33.7	639	35.7
P2Y12 inhibitor	615	33.7	639	35.7
Statin	619	34.0	640	35.7
ACE inhibitor/ARB	624	34.2	647	36.1
Beta-blocker	625	34.3	644	35.9
Calcium channel blocker	625	34.3	646	36.0
Insulin	615	33.7	640	35.7
Antidiabetic	615	33.7	640	35.7
Antidepressant	615	33.7	640	35.7
Established biomarkers				
eGFR*	15	0.8	6	0.3
Creatinine	15	0.8	6	0.3
CK	108	5.9	55	3.1
hsTnT	232	12.7	80	4.5

hsCRP	226	12.4	79	4.4
NT-proBNP	247	13.5	97	5.4
Leukocytes	181	9.9	21	1.2
Lymphocytes	194	10.6	903	50.4
Neutrophils	193	10.6	401	22.4
Neutrophil-to-lymphocyte ratio	204	11.2	916	51.1
HbA1c	394	21.6	1353	75.7
HDL-C	28	1.5	115	6.4
LDL-C	28	1.5	123	6.9
Triglycerides	21	1.2	117	6.5
Emerging biomarkers				
sLOX-1	441	24.2	531	29.6
cDPP3	87	4.8	108	6.0
PENK	88	4.8	109	6.1
PAM	91	5.0	113	6.3
ADM	89	4.9	109	6.1
ADM-Gly	117	6.4	145	8.1

*The estimated glomerular filtration rate (eGFR) was calculated using the Chronic Kidney Disease Epidemiology Collaboration (CKD-EPI) 2021 equation.[75] ADM, adrenomedullin; ADM-Gly, glycine-extended ADM; ACE, angiotensin-converting enzyme; ACS, acute coronary syndrome; ARB, angiotensin II receptor blocker; BMI, body mass index; CABG, coronary artery bypass grafting; CK, creatine kinase; cDPP3, circulating dipeptidyl peptidase 3; ECG, electrocardiogram; HbA1c, hemoglobin A1c; HDL-C, high-density lipoprotein cholesterol; hsCRP, high-sensitivity C-reactive protein; hsTnT, high-sensitivity troponin T; LDL-C, low-density lipoprotein cholesterol; LVEF, left ventricular ejection fraction; NT-proBNP, N-terminal pro B-type natriuretic peptide; PAM, peptidylglycine α-amidating monooxygenase; PENK, proenkephalin; PCI, percutaneous coronary intervention; sLOX-1, soluble lectin-like oxidised low-density lipoprotein receptor 1; TTS, takotsubo syndrome

Table S8: Completeness of BioTAK scocre variables

	All variables of the BioTAK score available	At least one variable of the BioTAK score missing
Development cohort	1302/1823 (71.4%)	521/1823 (28.6%)
Validation cohort	1141/1792 (63.7%)	651/1792 (36.3%)

The table summarises the number of patients in the development and validation cohorts with all variables available and the number of patients with at least one variable missing required for calculating the BioTAK score.

Table S9: Diagnostic accuracy across clinical subgroups

		Rule-out				Rule-in			
		Low-probability threshold		Very-low-probability threshold		High-probability threshold		Very-high-probability threshold	
		Sensitivity (95% CI)	NPV (95% CI)	Sensitivity (95% CI)	NPV (95% CI)	Specificity (95% CI)	PPV (95% CI)	Specificity (95% CI)	PPV (95% CI)
**Development cohort**									
All patients		95.7 (88.0–98.5)	99.8 (99.4–99.9)	98.6 (92.2–99.7)	99.9 (99.5–100)	99.0 (98.4–99.3)	59.1 (44.4–72.3)	99.9 (99.7–100)	88.9 (56.5–98.0)
Age	≤ 65 years	100 (83.9–100)	100 (99.6–100)	100 (83.9–100)	100 (99.5–100)	99.6 (98.9–99.8)	73.3 (48.1–89.1)	99.9 (99.4–100)	83.3 (43.7–96.7)
	> 65 years	93.9 (83.5–97.9)	99.6 (98.7–99.9)	98.0 (89.3–99.6)	99.8 (98.8–100)	98.3 (97.1–99.0)	51.7 (34.4–68.6)	100 (99.5–100)	100 (43.9–100)
Sex	Female	98.4 (91.5–99.7)	99.5 (97.1–99.9)	100 (94.0–100)	100 (94.0–100)	94.7 (91.8–96.6)	59.1 (44.4–72.3)	99.7 (98.3–100)	88.9 (56.6–98.0)
	Male*	66.7 (30.0–90.3)	99.9 (99.5–99.9)	83.3 (43.7–97.0)	99.9 (99.5–100)	100 (99.7–100)	†	100 (99.7–100)	†
ST-elevation	No	97.6 (87.7–99.6)	99.8 (99.1–99.9)	100 (91.6–100)	100 (99.1–100)	98.2 (96.9–98.9)	58.1 (40.8–73.6)	99.9 (99.2–100)	83.3 (43.7–97.0)
	Yes	92.6 (76.6–97.9)	99.8 (99.2–99.9)	96.3 (81.7–99.3)	99.9 (99.3–100)	99.5 (98.9–99.8)	61.5 (33.5–82.3)	100 (99.6–100)	100 (43.9–100)
Diabetes	No	94.8 (85.9–98.2)	99.8 (99.3–99.9)	98.3 (90.9–99.7)	99.9 (99.4–100)	99.2 (98.6–99.5)	63.6 (46.6–77.8)	99.9 (99.6–100)	85.7 (48.7–97.4)
	Yes	100 (74.0–100)	100 (98.0–100)	100 (74.1–100)	100 (98.0–100)	98.0 (95.6–99.1)	45.5 (21.3–72.0)	100 (98.7–100)	100 (34.2–100)
Hypertension	No	95.8 (79.8–99.3)	99.9 (99.2–99.9)	100 (86.2–100)	100 (99.3–100)	99.7 (99.1–99.9)	80.0 (49.0–94.3)	100 (99.5–100)	100 (34.2–100)
	Yes	95.6 (85.2–98.8)	99.8 (99.2–99.9)	97.8 (88.4–99.6)	99.8 (99.1–100)	98.4 (97.4–99.0)	52.9 (36.7–65.8)	99.9 (99.4–100)	85.7 (48.7–97.4)
Dyslipidaemia	No	98.0 (89.5–99.6)	99.8 (98.9–100)	100 (92.9–100)	100 (99.1–100)	98.6 (97.3–99.3)	69.2 (50.0–83.5)	100 (99.4–100)	100 (43.9–100)
	Yes	89.5 (58.5–97.1)	99.8 (99.3–99.9)	94.7 (75.4–99.1)	99.9 (99.3–100)	99.2 (98.4–99.5)	44.4 (24.6–66.3)	99.9 (99.5–100)	83.3 (43.7–97.0)
Evidence of pre-existing CAD	No	95.2 (86.7–98.3)	99.8 (99.3–99.9)	98.4 (91.4–99.7)	99.9 (99.5–100)	98.9 (98.3–99.3)	60.0 (44.6–73.7)	99.9 (99.7´6–100)	87.5 (52.9–97.8)
	Yes*	100 (64.6–100)	100 (98.4–100)	100 (64.6–100)	100 (97.7–100)	99.3 (97.3–99.8)	50.0 (15.0–85.0)	100 (98.6–100)	100 (20.7–100)

**Validation cohort**									
All patients		84.4 (74.7–90.9)	99.2 (98.7–99.6)	93.5 (85.7–97.2)	99.6 (99.0–99.9)	98.7 (98.0–99.1)	61.7 (49.0–72.9)	99.8 (99.5–99.9)	57.1 (25.1–84.2)
Age	≤ 65 years	73.9 (53.5–87.5)	99.3 (98.5–99.7)	91.3 (73.2–97.6)	99.7 (99.0–99.9)	99.7 (99.1–99.9)	75.0 (46.8–91.1)	99.9 (99.4–99.9)	†
	> 65 years	88.9 (77.8–94.8)	99.1 (98.1–99.6)	94.4 (84.9–98.1)	99.3 (97.9–99.8)	97.5 (96.1–98.3)	58.3 (44.3–71.2)	99.8 (99.1–99.9)	66.7 (30.0–90.3)
Sex	Female	93.9 (85.4–97.6)	98.1 (95.2–99.3)	97.0 (89.6–99.0)	96.1 (86.8–99.0)	93.7 (90.7–95.8)	61.7 (49.0–72.9)	99.2 (97.6–99.7)	57.1 (25.0–84.2)
	Male	27.3 (9.8–56.6)	99.4 (98.2–99.7)	72.7 (43.4–90.3)	99.7 (99.2–99.9)	100 (99.7–100)	†	100 (99.7–100)	†
ST-elevation	No	88.9 (77.8–94.8)	99.2 (98.3–99.6)	94.4 (84.9–98.1)	99.5 (98.4–99.8)	98.4 (97.3–99.0)	65.0 (49.5–77.9)	99.7 (99.0–99.9)	50.0 (18.8–81.2)
	Yes	73.9 (53.5–87.5)	99.2 (98.3–99.6)	91.3 (73.0–97.6)	99.7 (98.8–99.9)	99.0 (98.0–99.4)	55.0 (34.2–74.2)	100 (99.6–100)	100 (20.7–100)
Diabetes	No	84.5 (74.4–91.1)	99.1 (98.5–99.5)	93.0 (84.6–97.0)	99.5 (98.8–99.8)	98.9 (98.2–99.3)	68.6 (55.0–79.7)	99.9 (99.6–99.9)	80.0 (37.5–96.4)
	Yes*	83.3 (43.7–97.0)	99.6 (98.0–99.9)	100 (61.0–100)	100 (98.1–100)	97.7 (95.3–98.9)	22.2 (6.3–54.7)	99.3 (97.6–99.8)	†
Hypertension	No	75.0 (58.9–86.2)	98.8 (97.7–99.4)	88.9 (74.7–95.6)	99.3 (98.3–99.7)	99.2 (98.4–99.7)	66.7 (43.8–83.7)	99.9 (99.3–99.9)	50.0 (9.5–91.0)
	Yes	92.7 (80.6–97.5)	99.6 (98.9–99.9)	97.6 (87.4–99.6)	99.8 (99.0–100)	98.2 (97.1–98.8)	59.5 (44.5–73.0)	99.8 (99.2-99.9)	60.0 (23.1–88.2)
Dyslipidaemia	No	80.9 (67.5–89.6)	98.5 (97.3–99.2)	89.4 (77.4–95.4)	98.9 (97.4–99.5)	98.7 (97.5–99.3)	71.9 (54.6–84.4)	99.9 (99.2–99.9)	66.7 (20.8–93.9)
	Yes	90.0 (74.4–96.5)	99.7 (99.1–99.9)	100 (88.6–100)	100 (99.4–100)	98.6 (97.7–99.2)	50.0 (32.6–67.4)	99.8 (99.3–99.9)	50.0 (15.0–85.0)
Evidence of pre-existing CAD	No	86.3 (76.6–92.4)	99.2 (98.6–99.6)	93.2 (85.0–97.0)	99.5 (98.8–99.8)	99.1 (98.4-99.5)	73.4 (59.7–83.8)	99.9 (99.5–99.9)	66.7 (30.0–90.3)
	Yes*	50.0 (15.0–85.0)	99.3 (97.4–99.8)	100 (51.0–100)	100 (97.9–100)	96.9 (94.4–98.3)	9.1 (1.6–37.7)	99.7 (98.3–99.9)	†

Values represent percentages. † Value could not be derived because no true positive result was observed in this subgroup. *less than 10 Takotsubo diagnoses in subgroup; evidence of preexisting CAD was defined as recorded prior percutaneous coronary intervention, coronary artery bypass grafting, or myocardial infarction. CAD, coronary artery disease

Table S10: Diagnostic accuracy across different prevalence values

Prevalence of TTS	Rule-out				Rule-in			
	Low-probability threshold[&]		Very-low-probability threshold[§]		High-probability threshold[#]		Very-high-probability threshold[¶]	
	Proportion of all patients ruled out	NPV	Proportion of all patients ruled out	NPV	Proportion of all patients ruled in	PPV	Proportion of all patients ruled in	PPV
2.0 %	87.9	99.6	64.9	99.8	2.3	42.2	0.3	37.7
2.5 %	87.5	99.6	64.6	99.7	2.5	47.9	0.3	43.2
3.0 %	87.2	99.5	64.3	99.7	2.7	52.6	0.3	47.9
3.5 %	86.8	99.4	64.0	99.6	3.0	56.5	0.4	51.9
4.0 %	86.4	99.3	63.7	99.6	3.2	59.9	0.4	55.3
4.3 %*	86.2	99.2	63.5	99.6	3.3	61.7	0.4	57.1
4.5 %	86.1	99.2	63.4	99.5	3.4	62.8	0.4	58.3
5.0 %	85.7	99.1	63.1	99.5	3.7	65.3	0.4	61.0
5.5 %	85.3	99.0	62.8	99.4	3.9	67.6	0.5	63.3

* Prevalence of takotsubo syndrome (TTS) in the external validation cohort. Values represent percentages.

& Negative predictive values (NPVs) and the proportions ruled out were calculated based on the sensitivity (84.4%) and the specificity (89.4%) of the low-probability threshold of the BioTAK score in the external validation cohort assuming different prevalence values for TTS in the population.

§ NPVs and the proportions ruled out were calculated based on the sensitivity (93.5%) and the specificity (66.1%) of the very-low-probability threshold of the BioTAK score in the external validation cohort assuming different prevalence values for TTS in the population.

# Positive predictive values (PPVs) and the proportions ruled in were calculated based on the specificity (98.7%) and the sensitivity (48.1%) of the high-probability threshold of the BioTAK score in the external validation cohort assuming different prevalence values for TTS in the population.

¶ PPVs and the proportions ruled in were calculated based on the specificity (99.8%) and the sensitivity (5.2%) of the very-high-probability threshold of the BioTAK score in the external validation cohort assuming different prevalence values for TTS in the population.

Figure S1: Flow-chart of the study

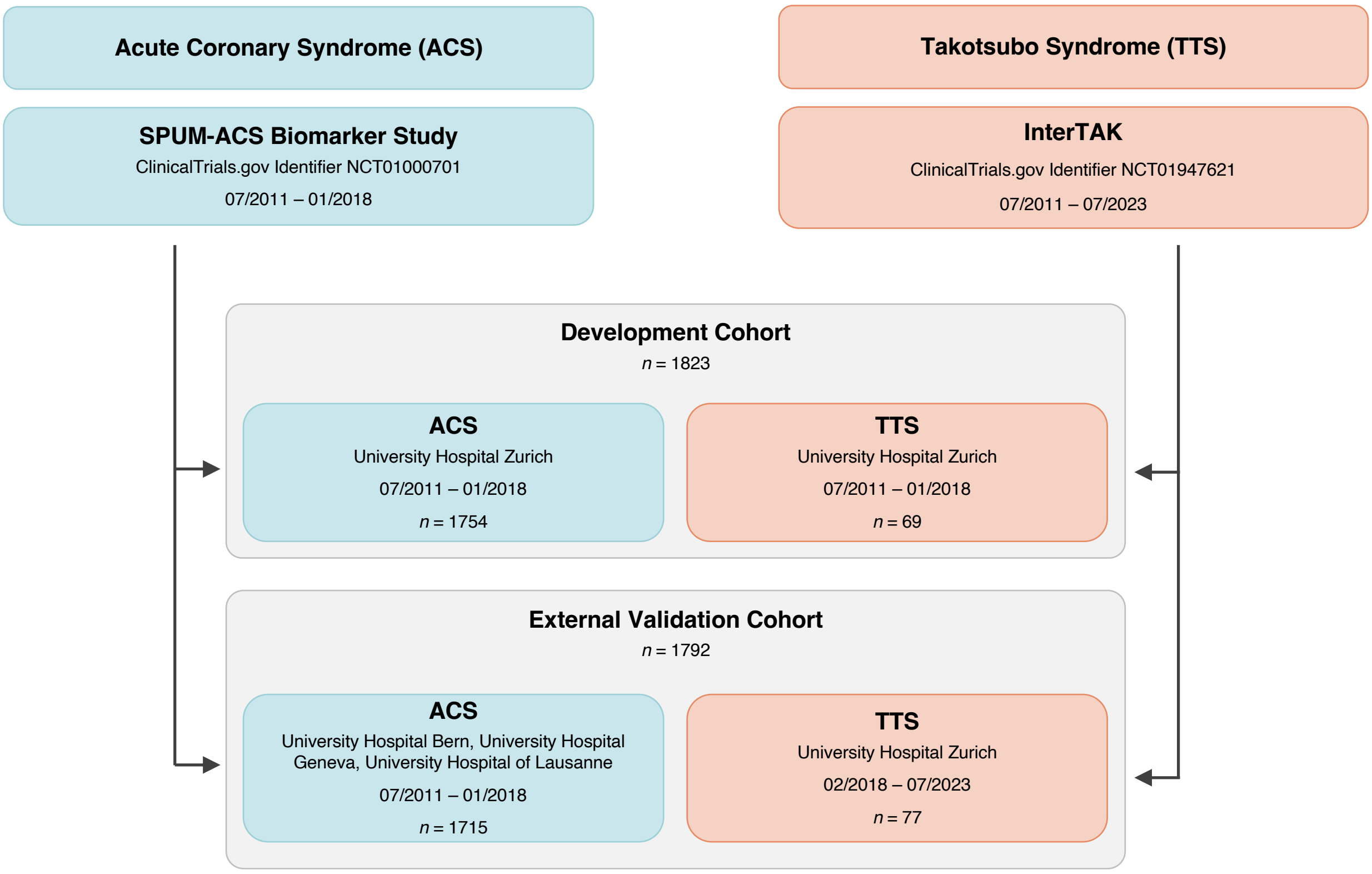

Figure S2: Violin plots of biomarker levels in development and validation cohorts

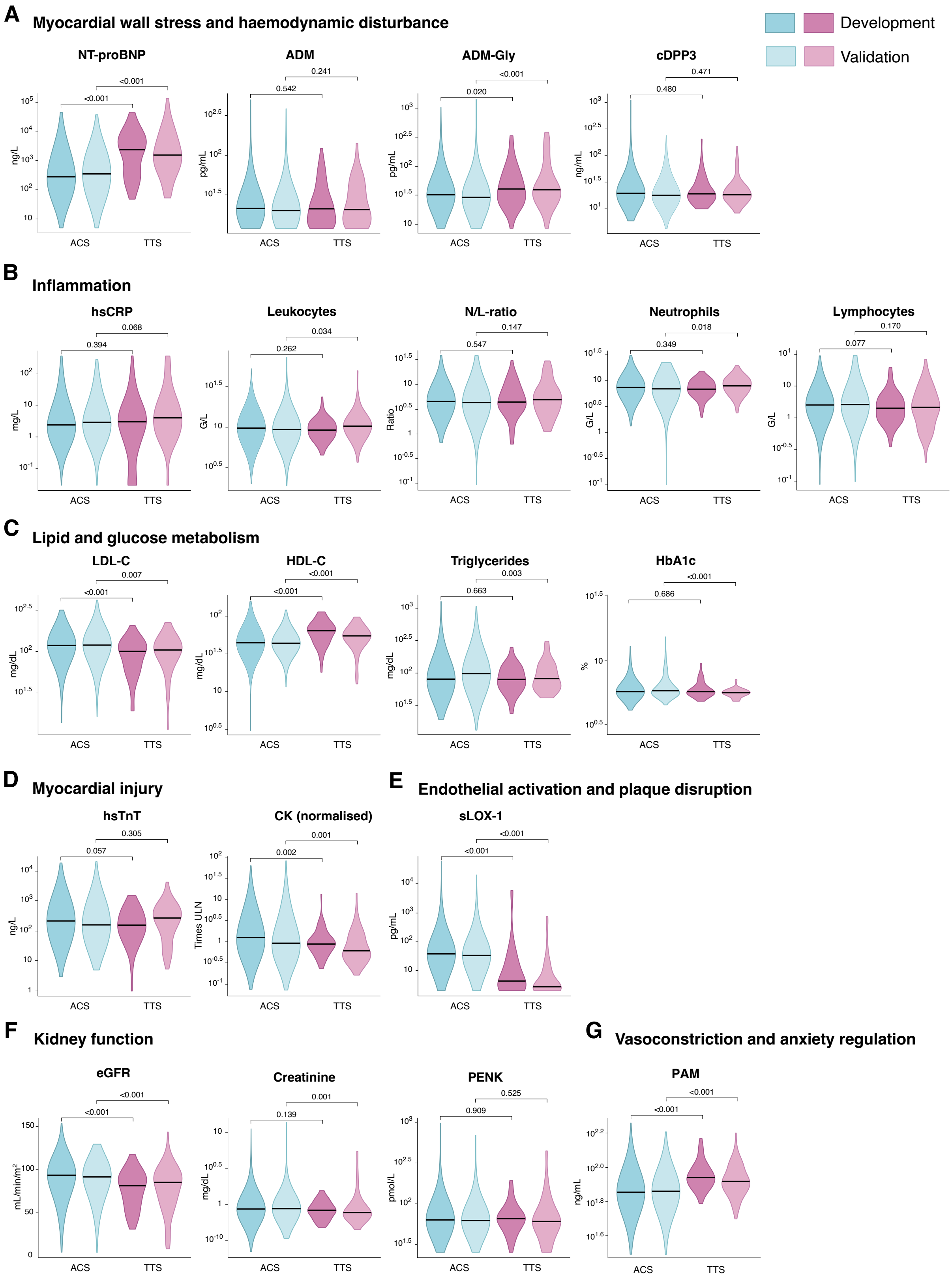

Violin plots show the distribution of markers related to (A) myocardial wall stress and haemodynamic disturbance, (B) inflammation, (C) lipid and glucose metabolism, (D) myocardial injury, (E) endothelial activation and plaque disruption, (F) kidney function, (G) vasoconstriction and anxiety regulation in patients with acute coronary syndrome (ACS) and takotsubo syndrome (TTS). Groups were compared using the Mann-Whitey-U test.

ADM, adrenomedullin; ADM-Gly, glycine-extended ADM; CK, creatine kinase; DPP3, dipeptidyl peptidase 3; eGFR, estimated glomerular filtration rate; HbA1c, hemoglobin A1c; HDL-C, high-density lipoprotein cholesterol; hs-CRP, high-sensitivity C-reactive protein; hs-TnT, high-sensitivity troponin T; LDL-C, low-density lipoprotein cholesterol; N/L, neutrophil/lymphocyte; NT-proBNP, N-terminal pro B-type natriuretic peptide; PAM, peptidylglycine α-amidating monooxygenase; PENK, proenkephalin; sLOX, soluble Lectin-like oxidised low-density lipoprotein; ULN, upper limit of normal

Figure S3: Calibration plot in development cohort

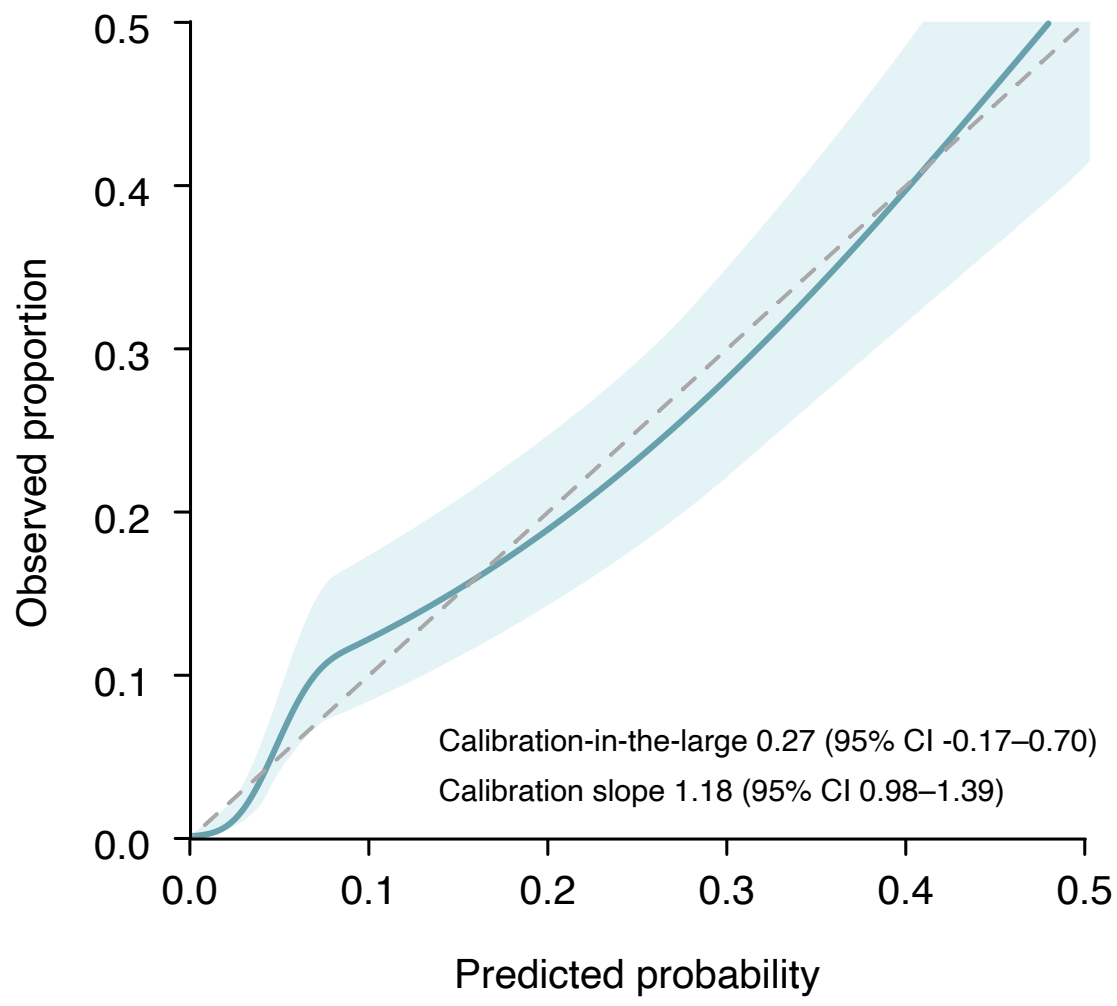

Calibration curve of the BioTAK score in the development cohort. The dashed diagonal line indicates ideal calibration. The solid line shows the smoothed calibration curves with the colour band signifying its 95% confidence interval (CI). The smoothed calibration curve was constructed using a restricted cubic spline function with 5 knots.

Figure S4: Bias-corrected calibration curves

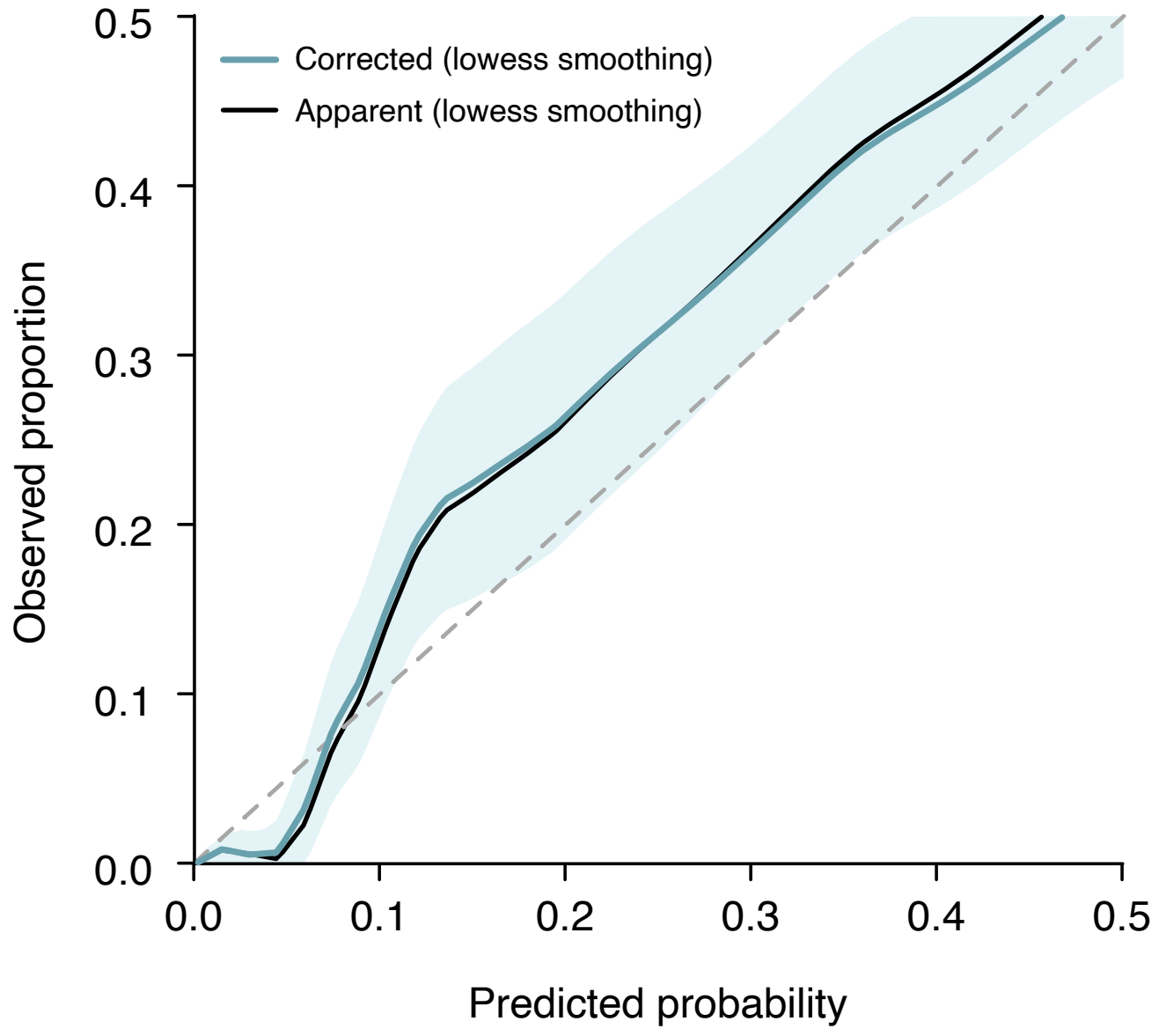

Bias (optimism)-corrected and apparent calibration curves of the BioTAK score in the development cohort. The dashed diagonal line indicates ideal calibration. The solid lines show the smoothed calibration curves with the colour band signifying its 95% confidence interval (CI). The smoothed calibration curve was constructed using the locally weighted scatterplot smoothing function.

Figure S5: Decision curves of the BioTAK score in development and validation cohorts

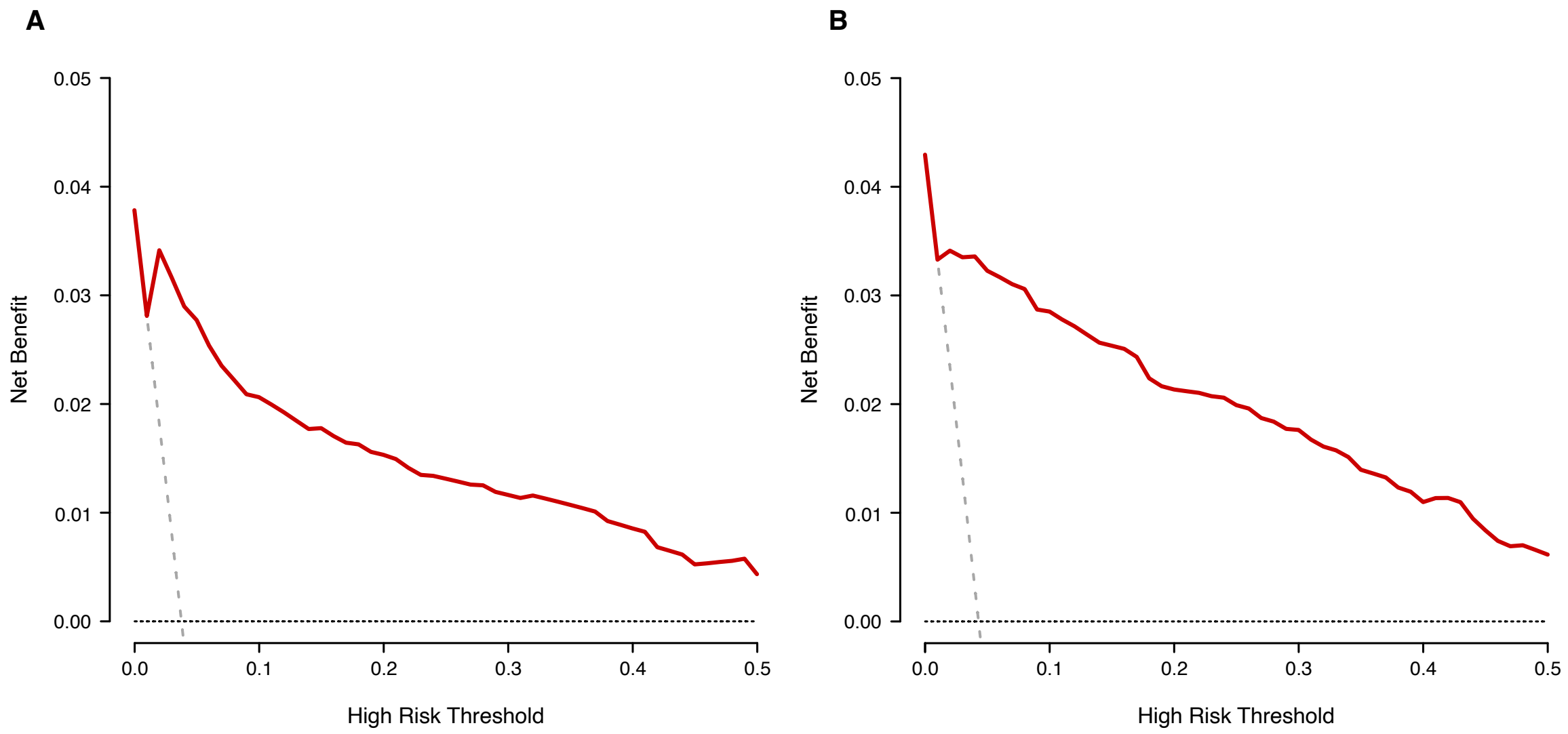

Plots display the net benefit of the BioTAK score across a range of decision thresholds (A) in the development cohort and (B) in the validation cohort. Horizontal black dotted lines represent a treat-none-scenario, whereas oblique grey dashed lines represent a treat-all-scenario.

Figure S6: Nomogram for calculation of the BioTAK score

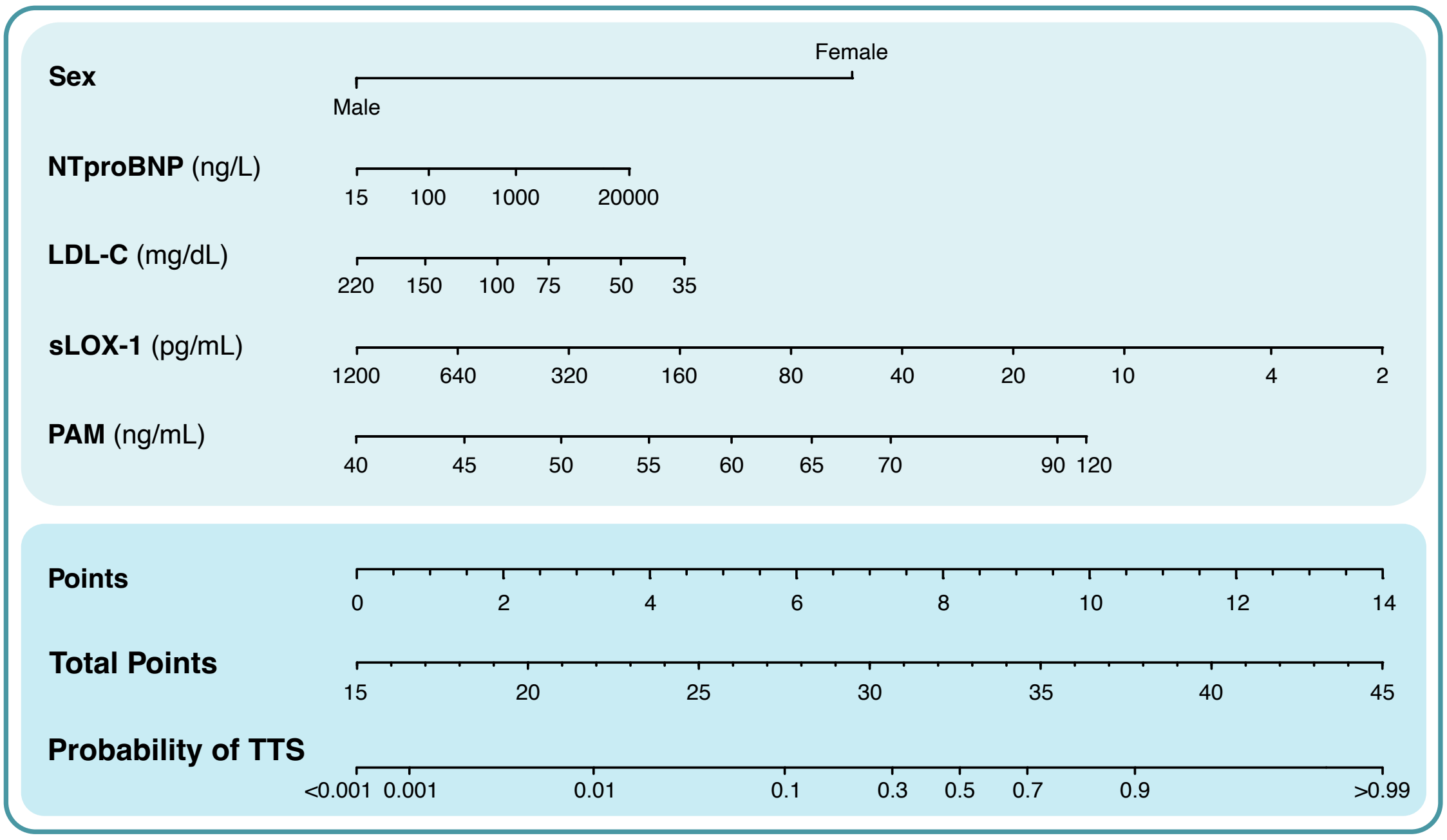

Total point values below 15 and above 45 correspond to predicted probabilities of TTS of <0.001 and >0.99, respectively.
LDL-C, low-density lipoprotein cholesterol; NT-proBNP, N-terminal pro B-type natriuretic peptide; PAM, peptidylglycine α-amidating monooxygenase; sLOX-1, soluble lectin-like oxidised low-density lipoprotein receptor-1; TTS, takotsubo syndrome

Figure S7: Stratification of patients with possible takotsubo syndrome in the validation cohort

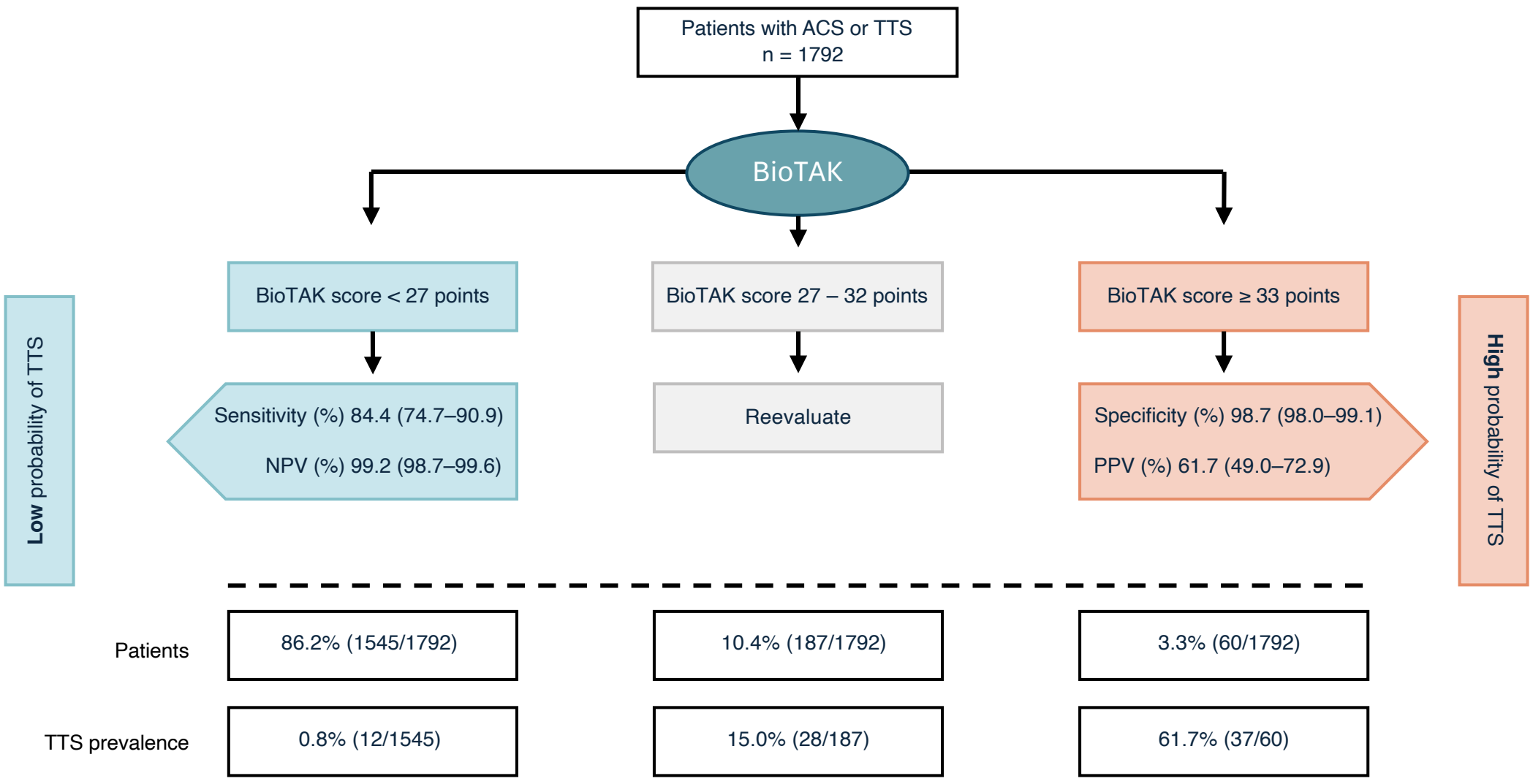

ACS, acute coronary syndrome; NPV, negative predictive value; PPV, positive predictive value; TTS, takotsubo syndrome.

Figure S8: Stratification of patients with possible takotsubo syndrome presenting with ST-segment elevation

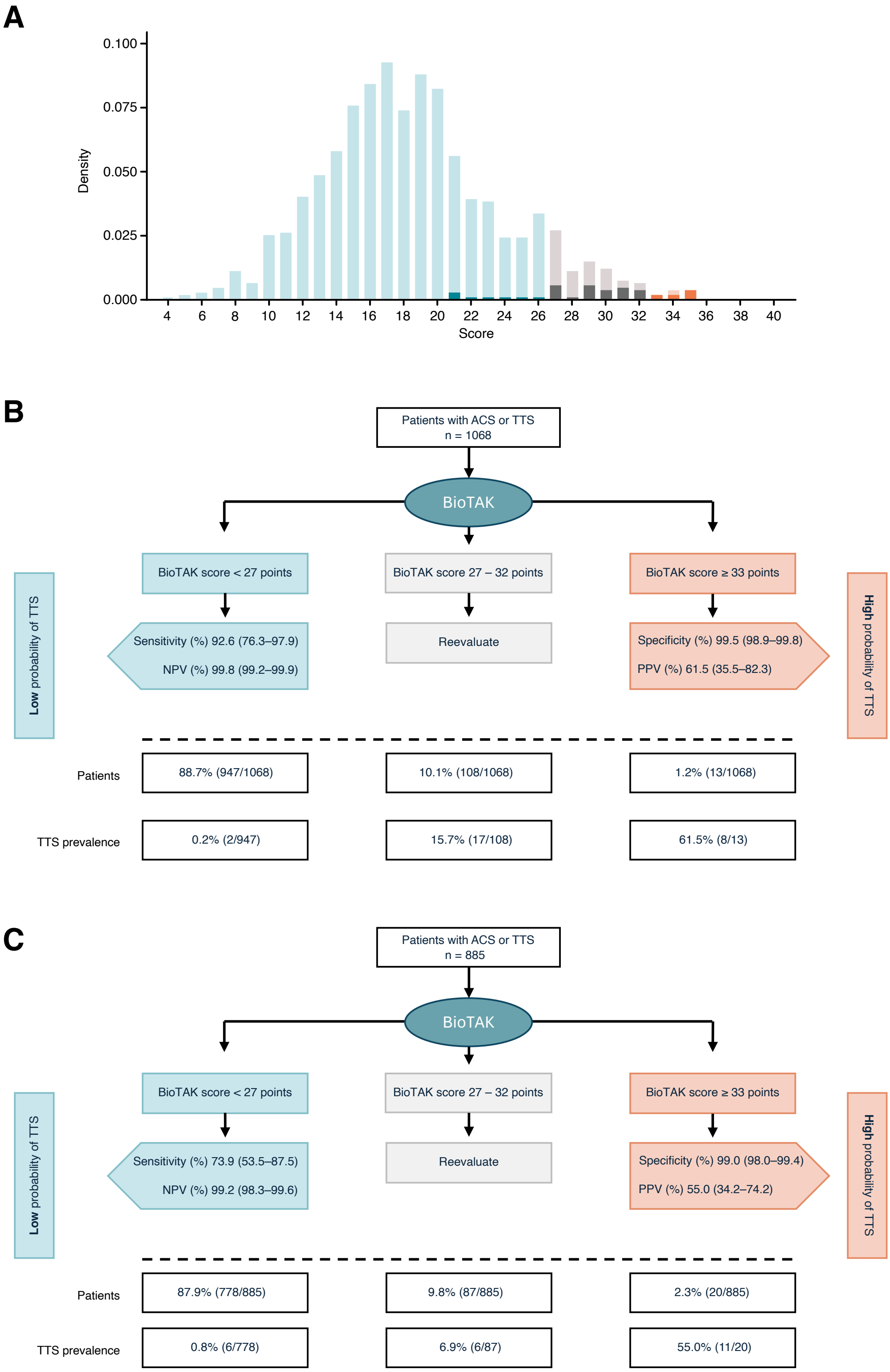

(A) Distribution of score values stratified by diagnostic outcome in patients presenting with ST-elevation in the development cohort and stratification of patients presenting with ST-elevation using the BioTAK diagnostic score and its rule-in and rule-out thresholds (B) in the development cohort and (C) in the validation cohort. Darker bar segments indicate patients with a final diagnosis of takotsubo syndrome. Colours indicate the pre-specified low-probability (<27 points), intermediate-probability (27 – 32 points) and high-probability (≥33

points) categories. ACS, acute coronary syndrome; NPV, negative predictive value; PPV, positive predictive value; TTS, takotsubo syndrome.

Figure S9: Stratification of patients with possible takotsubo syndrome presenting without ST-segment elevation

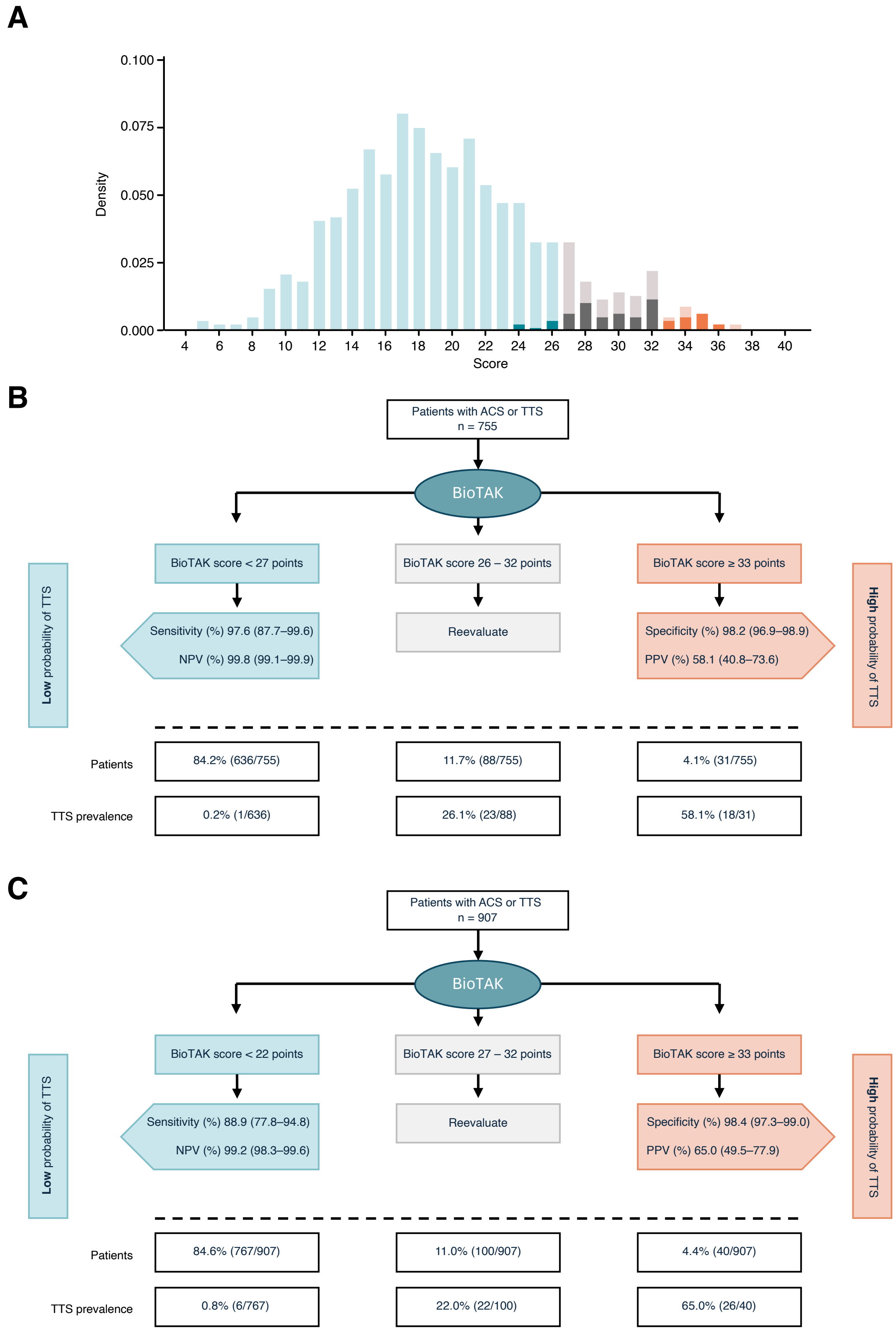

(A) Distribution of score values stratified by diagnostic outcome in patients presenting without ST-elevation in the development cohort and stratification of patients presenting without ST-elevation using the BioTAK diagnostic score and its rule-in and rule-out thresholds (B) in the development cohort and (C) in the validation cohort. Darker bar segments indicate patients with a final diagnosis of takotsubo syndrome. Colours indicate the pre-specified low-probability (<27 points), intermediate-probability (27 – 32 points) and high-probability (≥33

points) categories. ACS, acute coronary syndrome; NPV, negative predictive value; PPV, positive predictive value; TTS, takotsubo syndrome.

Figure S10: STARD Checklist

Section & Topic	No	Item	Reported on page #
**TITLE OR ABSTRACT**			
	**1**	Identification as a study of diagnostic accuracy using at least one measure of accuracy<br>(such as sensitivity, specificity, predictive values, or AUC)	2
**ABSTRACT**			
	**2**	Structured summary of study design, methods, results, and conclusions<br>(for specific guidance, see STARD for Abstracts)	2,3
**INTRODUCTION**			
	**3**	Scientific and clinical background, including the intended use and clinical role of the index test	6
	**4**	Study objectives and hypotheses	6, 7
**METHODS**			
Study design	**5**	Whether data collection was planned before the index test and reference standard were performed (prospective study) or after (retrospective study)	7
Participants	**6**	Eligibility criteria	7
	**7**	On what basis potentially eligible participants were identified<br>(such as symptoms, results from previous tests, inclusion in registry)	7, 8
	**8**	Where and when potentially eligible participants were identified (setting, location and dates)	7, 8
	**9**	Whether participants formed a consecutive, random or convenience series	7
Test methods	**10a**	Index test, in sufficient detail to allow replication	9–11, Suppl.
	**10b**	Reference standard, in sufficient detail to allow replication	10
	**11**	Rationale for choosing the reference standard (if alternatives exist)	10
	**12a**	Definition of and rationale for test positivity cut-offs or result categories of the index test, distinguishing pre-specified from exploratory	10
	**12b**	Definition of and rationale for test positivity cut-offs or result categories of the reference standard, distinguishing pre-specified from exploratory	10
	**13a**	Whether clinical information and reference standard results were available to the performers/readers of the index test	10
	**13b**	Whether clinical information and index test results were available to the assessors of the reference standard	10
Analysis	**14**	Methods for estimating or comparing measures of diagnostic accuracy	10, 11, Suppl.
	**15**	How indeterminate index test or reference standard results were handled	Suppl.
	**16**	How missing data on the index test and reference standard were handled	10, 11, Suppl.
	**17**	Any analyses of variability in diagnostic accuracy, distinguishing pre-specified from exploratory	10
	**18**	Intended sample size and how it was determined	10, 11
**RESULTS**			
Participants	**19**	Flow of participants, using a diagram	Suppl.
	**20**	Baseline demographic and clinical characteristics of participants	11, Table 1
	**21a**	Distribution of severity of disease in those with the target condition	11, Table 1
	**21b**	Distribution of alternative diagnoses in those without the target condition	11, Table 1
	**22**	Time interval and any clinical interventions between index test and reference standard	11
Test results	**23**	Cross tabulation of the index test results (or their distribution) by the results of the reference standard	Figure 3, Suppl.
	**24**	Estimates of diagnostic accuracy and their precision (such as 95% confidence intervals)	12-14, Suppl.
	**25**	Any adverse events from performing the index test or the reference standard	N/A
**DISCUSSION**			

	**26**	Study limitations, including sources of potential bias, statistical uncertainty, and generalisability	16–18
	**27**	Implications for practice, including the intended use and clinical role of the index test	14–16, 18
**OTHER INFORMATION**			
	**28**	Registration number and name of registry	7, 21
	**29**	Where the full study protocol can be accessed	21
	**30**	Sources of funding and other support; role of funders	18, 19

Figure S11: TRIPOD Checklist

TRIPOD Checklist: Prediction Model Development

Section/Topic	Item	Checklist Item	Page
**Title and abstract**			
Title	1	Identify the study as developing and/or validating a multivariable prediction model, the target population, and the outcome to be predicted.	1
Abstract	2	Provide a summary of objectives, study design, setting, participants, sample size, predictors, outcome, statistical analysis, results, and conclusions.	2
**Introduction**			
Background and objectives	3a	Explain the medical context (including whether diagnostic or prognostic) and rationale for developing or validating the multivariable prediction model, including references to existing models.	6
	3b	Specify the objectives, including whether the study describes the development or validation of the model or both.	6, 7
**Methods**			
Source of data	4a	Describe the study design or source of data (e.g., randomized trial, cohort, or registry data), separately for the development and validation data sets, if applicable.	7
	4b	Specify the key study dates, including start of accrual; end of accrual; and, if applicable, end of follow-up.	7
Participants	5a	Specify key elements of the study setting (e.g., primary care, secondary care, general population) including number and location of centres.	7, 8
	5b	Describe eligibility criteria for participants.	7,8
	5c	Give details of treatments received, if relevant.	7,8
Outcome	6a	Clearly define the outcome that is predicted by the prediction model, including how and when assessed.	8
	6b	Report any actions to blind assessment of the outcome to be predicted.	N/A
Predictors	7a	Clearly define all predictors used in developing or validating the multivariable prediction model, including how and when they were measured.	9, Suppl.
	7b	Report any actions to blind assessment of predictors for the outcome and other predictors.	N/A
Sample size	8	Explain how the study size was arrived at.	11, Suppl.
Missing data	9	Describe how missing data were handled (e.g., complete-case analysis, single imputation, multiple imputation) with details of any imputation method.	10, Suppl.
Statistical analysis methods	10a	Describe how predictors were handled in the analyses.	9, Suppl.
	10b	Specify type of model, all model-building procedures (including any predictor selection), and method for internal validation.	9, Suppl.
	10d	Specify all measures used to assess model performance and, if relevant, to compare multiple models.	9, 10 , Suppl.
Risk groups	11	Provide details on how risk groups were created, if done.	10
**Results**			
Participants	13a	Describe the flow of participants through the study, including the number of participants with and without the outcome and, if applicable, a summary of the follow-up time. A diagram may be helpful.	Suppl.
	13b	Describe the characteristics of the participants (basic demographics, clinical features, available predictors), including the number of participants with missing data for predictors and outcome.	11, Table 1
Model development	14a	Specify the number of participants and outcome events in each analysis.	Table 1, Suppl.
	14b	If done, report the unadjusted association between each candidate predictor and outcome.	Suppl.
Model specification	15a	Present the full prediction model to allow predictions for individuals (i.e., all regression coefficients, and model intercept or baseline survival at a given time point).	11, Suppl.
	15b	Explain how to the use the prediction model.	11
Model performance	16	Report performance measures (with CIs) for the prediction model.	12–13
**Discussion**			
Limitations	18	Discuss any limitations of the study (such as nonrepresentative sample, few events per predictor, missing data).	17–18
Interpretation	19b	Give an overall interpretation of the results, considering objectives, limitations, and results from similar studies, and other relevant evidence.	14–16
Implications	20	Discuss the potential clinical use of the model and implications for future research.	18
**Other information**			
Supplementary information	21	Provide information about the availability of supplementary resources, such as study protocol, Web calculator, and data sets.	21
Funding	22	Give the source of funding and the role of the funders for the present study.	18, 19

We recommend using the TRIPOD Checklist in conjunction with the TRIPOD Explanation and Elaboration document.

References

1. Wenzl FA, Bruno F, Kraler S, et al. Dipeptidyl peptidase 3 plasma levels predict cardiogenic shock and mortality in acute coronary syndromes. *Eur Heart J* 2023; **44**(38): 3859-71.
2. Kraler S, Wenzl FA, Georgiopoulos G, et al. Soluble lectin-like oxidized low-density lipoprotein receptor-1 predicts premature death in acute coronary syndromes. *Eur Heart J* 2022; **43**(19): 1849-60.
3. Ghadri JR, Cammann VL, Jurisic S, et al. A novel clinical score (InterTAK Diagnostic Score) to differentiate takotsubo syndrome from acute coronary syndrome: results from the International Takotsubo Registry. *Eur J Heart Fail* 2017; **19**(8): 1036-42.
4. Dagrenat C, Von Hunolstein JJ, Matsushita K, et al. Value of Cardiac Biomarkers in the Early Diagnosis of Takotsubo Syndrome. *J Clin Med* 2020; **9**(9).
5. Simonsson M, Winell H, Olsson H, et al. Development and Validation of a Novel Risk Score for In-Hospital Major Bleeding in Acute Myocardial Infarction:-The SWEDEHEART Score. *J Am Heart Assoc* 2019; **8**(5): e012157.
6. Batra G, Lindback J, Becker RC, et al. Biomarker-Based Prediction of Recurrent Ischemic Events in Patients With Acute Coronary Syndromes. *J Am Coll Cardiol* 2022; **80**(18): 1735-47.
7. Wenzl FA, Wang P, Arrigo M, et al. Proenkephalin Improves Cardio-Renal Risk Prediction in Acute Coronary Syndromes: The KID-ACS Score. *Eur Heart J* 2024.
8. Hijazi Z, Oldgren J, Lindback J, et al. The novel biomarker-based ABC (age, biomarkers, clinical history)-bleeding risk score for patients with atrial fibrillation: a derivation and validation study. *Lancet* 2016; **387**(10035): 2302-11.
9. Fabritz L, Al-Taie C, Borof K, et al. Biomarker-based prediction of sinus rhythm in atrial fibrillation patients: the EAST-AFNET 4 biomolecule study. *Eur Heart J* 2024; **45**(47): 5002-19.
10. Wang J, Gao W, Chen G, et al. Biomarker-based risk model to predict cardiovascular events in patients with acute coronary syndromes - Results from BIPass registry. *Lancet Reg Health West Pac* 2022; **25**: 100479.
11. Burkart KG, Brauer M, Aravkin AY, et al. Estimating the cause-specific relative risks of non-optimal temperature on daily mortality: a two-part modelling approach applied to the Global Burden of Disease Study. *Lancet* 2021; **398**(10301): 685-97.
12. Liu J, Liu T, Burkart KG, et al. Mortality burden attributable to high and low ambient temperatures in China and its provinces: Results from the Global Burden of Disease Study 2019. *Lancet Reg Health West Pac* 2022; **24**: 100493.
13. Harrell FE, Jr., Lee KL, Califf RM, Pryor DB, Rosati RA. Regression modelling strategies for improved prognostic prediction. *Stat Med* 1984; **3**(2): 143-52.
14. Hijazi Z, Oldgren J, Lindbäck J, et al. The novel biomarker-based ABC (age, biomarkers, clinical history)-bleeding risk score for patients with atrial fibrillation: a derivation and validation study. *Lancet* 2016; **387**(10035): 2302-11.
15. Wenzl FA, Kofoed KF, Simonsson M, et al. Extension of the GRACE score for non-ST-elevation acute coronary syndrome: a development and validation study in ten countries. *Lancet Digit Health* 2025; **7**(10): 100907.
16. Wenzl FA, Kraler S, Ambler G, et al. Sex-specific evaluation and redevelopment of the GRACE score in non-ST-segment elevation acute coronary syndromes in populations from the UK and Switzerland: a multinational analysis with external cohort validation. *Lancet* 2022; **400**(10354): 744-56.
17. Rothman KJ. Epidemiology: An Introduction: OUP USA; 2012.
18. Ercole A, Brinck V, George P, et al. Guidelines for Data Acquisition, Quality and Curation for Observational Research Designs (DAQCORD). *J Clin Transl Sci* 2020; **4**(4): 354-9.
19. Steinfeldt J, Buergel T, Loock L, et al. Neural network-based integration of polygenic and clinical information: development and validation of a prediction model for 10-year risk of major adverse cardiac events in the UK Biobank cohort. *Lancet Digit Health* 2022; **4**(2): e84-e94.
20. Forrest IS, Petrazzini BO, Duffy A, et al. Machine learning-based marker for coronary artery disease: derivation and validation in two longitudinal cohorts. *Lancet* 2023; **401**(10372): 215-25.
21. Petrazzini BO, Chaudhary K, Marquez-Luna C, et al. Coronary Risk Estimation Based on Clinical Data in Electronic Health Records. *J Am Coll Cardiol* 2022; **79**(12): 1155-66.
22. Wenzl FA, Ow KW, Velders MA, et al. Prediction of mortality, bleeding, and ischaemic events in patients with cancer and acute coronary syndrome: a model development and validation study. *Lancet* 2026; **407**(10527): 515-28.
23. Austin PC, Lee DS, Ko DT, White IR. Effect of Variable Selection Strategy on the Performance of Prognostic Models When Using Multiple Imputation. *Circ Cardiovasc Qual Outcomes* 2019; **12**(11): e005927.
24. van Buuren S, Groothuis-Oudshoorn K. mice: Multivariate Imputation by Chained Equations in R. *Journal of Statistical Software* 2011; **45**(3): 1 - 67.
25. Rubin DB. Multiple imputation for survey nonresponse. New York: Wiley; 1987.

26. Ceglarek U, Schellong P, Rosolowski M, et al. The novel cystatin C, lactate, interleukin-6, and N-terminal pro-B-type natriuretic peptide (CLIP)-based mortality risk score in cardiogenic shock after acute myocardial infarction. *Eur Heart J* 2021; **42**(24): 2344-52.
27. Faghri F, Brunn F, Dadu A, et al. Identifying and predicting amyotrophic lateral sclerosis clinical subgroups: a population-based machine-learning study. *Lancet Digit Health* 2022; **4**(5): e359-e69.
28. Lee DS, Wang CX, McAlister FA, et al. Factors associated with SARS-CoV-2 test positivity in long-term care homes: A population-based cohort analysis using machine learning. *Lancet Reg Health Am* 2022; **6**: 100146.
29. Sarvestany SS, Kwong JC, Azhie A, et al. Development and validation of an ensemble machine learning framework for detection of all-cause advanced hepatic fibrosis: a retrospective cohort study. *Lancet Digit Health* 2022; **4**(3): e188-e99.
30. Mohammad MA, Olesen KKW, Koul S, et al. Development and validation of an artificial neural network algorithm to predict mortality and admission to hospital for heart failure after myocardial infarction: a nationwide population-based study. *Lancet Digit Health* 2022; **4**(1): e37-e45.
31. De Filippo O, Cammann VL, Pancotti C, et al. Machine learning-based prediction of in-hospital death for patients with takotsubo syndrome: The InterTAK-ML model. *Eur J Heart Fail* 2023.
32. Chow SL, Maisel AS, Anand I, et al. Role of Biomarkers for the Prevention, Assessment, and Management of Heart Failure: A Scientific Statement From the American Heart Association. *Circulation* 2017; **135**(22): e1054-e91.
33. Nguyen TH, Neil CJ, Sverdlov AL, et al. N-terminal pro-brain natriuretic protein levels in takotsubo cardiomyopathy. *Am J Cardiol* 2011; **108**(9): 1316-21.
34. Ghadri JR, Wittstein IS, Prasad A, et al. International Expert Consensus Document on Takotsubo Syndrome (Part II): Diagnostic Workup, Outcome, and Management. *Eur Heart J* 2018; **39**(22): 2047-62.
35. Marino R, Struck J, Maisel AS, Magrini L, Bergmann A, Di Somma S. Plasma adrenomedullin is associated with short-term mortality and vasopressor requirement in patients admitted with sepsis. *Crit Care* 2014; **18**(1): R34.
36. Voors AA, Kremer D, Geven C, et al. Adrenomedullin in heart failure: pathophysiology and therapeutic application. *Eur J Heart Fail* 2019; **21**(2): 163-71.
37. Ter Maaten JM, Kremer D, Demissei BG, et al. Bio-adrenomedullin as a marker of congestion in patients with new-onset and worsening heart failure. *Eur J Heart Fail* 2019; **21**(6): 732-43.
38. Prigge ST, Mains RE, Eipper BA, Amzel LM. New insights into copper monooxygenases and peptide amidation: structure, mechanism and function. *Cell Mol Life Sci* 2000; **57**(8-9): 1236-59.
39. Hirayama N, Kitamura K, Imamura T, et al. Molecular forms of circulating adrenomedullin in patients with congestive heart failure. *J Endocrinol* 1999; **160**(2): 297-303.
40. Nishikimi T, Asakawa H, Iida H, et al. Different secretion patterns of two molecular forms of cardiac adrenomedullin in pressure- and volume-overloaded human heart failure. *J Card Fail* 2004; **10**(4): 321-7.
41. Pang X, Shimizu A, Kurita S, et al. Novel Therapeutic Role for Dipeptidyl Peptidase III in the Treatment of Hypertension. *Hypertension* 2016; **68**(3): 630-41.
42. Deniau B, Rehfeld L, Santos K, et al. Circulating dipeptidyl peptidase 3 is a myocardial depressant factor: dipeptidyl peptidase 3 inhibition rapidly and sustainably improves haemodynamics. *Eur J Heart Fail* 2020; **22**(2): 290-9.
43. Gamrekelashvili J, Kapanadze T, Han M, et al. Peptidases released by necrotic cells control CD8+ T cell cross-priming. *J Clin Invest* 2013; **123**(11): 4755-68.
44. Moady G, Yelin B, Sweid R, Atar S. C-Reactive Protein Can Predict Outcomes in Patients With Takotsubo Syndrome. *Int J Heart Fail* 2024; **6**(1): 28-33.
45. Lee S. The Relationship Between C-reactive Protein and Takotsubo Syndrome: An Old Riddle. *Int J Heart Fail* 2024; **6**(1): 34-5.
46. Swirski FK, Nahrendorf M. Leukocyte behavior in atherosclerosis, myocardial infarction, and heart failure. *Science* 2013; **339**(6116): 161-6.
47. Novo G, Arcari L, Madaudo C, et al. Blood Cell Subtype patterns in Takotsubo Syndrome and acute coronary syndrome: analysis from the GEIST registry. *Atherosclerosis* 2025; **411**: 120571.
48. Ference BA, Ginsberg HN, Graham I, et al. Low-density lipoproteins cause atherosclerotic cardiovascular disease. 1. Evidence from genetic, epidemiologic, and clinical studies. A consensus statement from the European Atherosclerosis Society Consensus Panel. *Eur Heart J* 2017; **38**(32): 2459-72.
49. Gordon T, Castelli WP, Hjortland MC, Kannel WB, Dawber TR. High density lipoprotein as a protective factor against coronary heart disease. The Framingham Study. *Am J Med* 1977; **62**(5): 707-14.
50. Castelli WP, Garrison RJ, Wilson PW, Abbott RD, Kalousdian S, Kannel WB. Incidence of coronary heart disease and lipoprotein cholesterol levels. The Framingham Study. *Jama* 1986; **256**(20): 2835-8.
51. Nordestgaard BG, Benn M, Schnohr P, Tybjaerg-Hansen A. Nonfasting triglycerides and risk of myocardial infarction, ischemic heart disease, and death in men and women. *Jama* 2007; **298**(3): 299-308.

52. Selvin E, Steffes MW, Zhu H, et al. Glycated hemoglobin, diabetes, and cardiovascular risk in nondiabetic adults. *N Engl J Med* 2010; **362**(9): 800-11.
53. Schweiger V, Di Vece D, Cammann VL, et al. Cardiac biomarkers for diagnosing Takotsubo syndrome. *Eur Heart J* 2024; **45**(25): 2254-8.
54. Templin C, Ghadri JR, Diekmann J, et al. Clinical Features and Outcomes of Takotsubo (Stress) Cardiomyopathy. *N Engl J Med* 2015; **373**(10): 929-38.
55. Brewster LM, Fernand J. Creatine kinase during non-ST-segment elevation acute coronary syndromes is associated with major bleeding. *Open Heart* 2020; **7**(2).
56. Li D, Patel AR, Klibanov AL, et al. Molecular imaging of atherosclerotic plaques targeted to oxidized LDL receptor LOX-1 by SPECT/CT and magnetic resonance. *Circ Cardiovasc Imaging* 2010; **3**(4): 464-72.
57. Hofmann A, Brunssen C, Wolk S, Reeps C, Morawietz H. Soluble LOX-1: A Novel Biomarker in Patients With Coronary Artery Disease, Stroke, and Acute Aortic Dissection? *J Am Heart Assoc* 2020; **9**(1): e013803.
58. Mentrup T, Theodorou K, Cabrera-Cabrera F, et al. Atherogenic LOX-1 signaling is controlled by SPPL2-mediated intramembrane proteolysis. *J Exp Med* 2019; **216**(4): 807-30.
59. Akhmedov A, Sawamura T, Chen CH, Kraler S, Vdovenko D, Lüscher TF. Lectin-like oxidized low-density lipoprotein receptor-1 (LOX-1): a crucial driver of atherosclerotic cardiovascular disease. *Eur Heart J* 2021; **42**(18): 1797-807.
60. Lindberg I, Glembotski CC. Physiological signaling in the absence of amidated peptides. *Proc Natl Acad Sci U S A* 2019; **116**(40): 19774-6.
61. Powers KG, Ma XM, Eipper BA, Mains RE. Identifying roles for peptidergic signaling in mice. *Proc Natl Acad Sci U S A* 2019; **116**(40): 20169-79.
62. Bäck N, Luxmi R, Powers KG, Mains RE, Eipper BA. Peptidylglycine α-amidating monooxygenase is required for atrial secretory granule formation. *Proc Natl Acad Sci U S A* 2020; **117**(30): 17820-31.
63. Santopinto JJ, Fox KA, Goldberg RJ, et al. Creatinine clearance and adverse hospital outcomes in patients with acute coronary syndromes: findings from the global registry of acute coronary events (GRACE). *Heart* 2003; **89**(9): 1003-8.
64. Granger CB, Goldberg RJ, Dabbous O, et al. Predictors of hospital mortality in the global registry of acute coronary events. *Arch Intern Med* 2003; **163**(19): 2345-53.
65. Ronco C, Bellomo R, Kellum JA. Acute kidney injury. *Lancet* 2019; **394**(10212): 1949-64.
66. Zarbock A, Nadim MK, Pickkers P, et al. Sepsis-associated acute kidney injury: consensus report of the 28th Acute Disease Quality Initiative workgroup. *Nat Rev Nephrol* 2023; **19**(6): 401-17.
67. Marino R, Struck J, Hartmann O, et al. Diagnostic and short-term prognostic utility of plasma pro-enkephalin (pro-ENK) for acute kidney injury in patients admitted with sepsis in the emergency department. *J Nephrol* 2015; **28**(6): 717-24.
68. Kraler S, Wenzl FA, Georgiopoulos G, et al. Soluble lectin-like oxidized low-density lipoprotein receptor-1 predicts premature death in acute coronary syndromes. *Eur Heart J* 2022; **43**(19): 1849-60.
69. Ilina Y, Kaufmann P, Melander O, Press M, Thuene K, Bergmann A. Immunoassay-based quantification of full-length peptidylglycine alpha-amidating monooxygenase in human plasma. *Sci Rep* 2023; **13**(1): 10827.
70. Weber J, Sachse J, Bergmann S, Sparwaßer A, Struck J, Bergmann A. Sandwich Immunoassay for Bioactive Plasma Adrenomedullin. *J Appl Lab Med* 2017; **2**(2): 222-33.
71. Kaufmann P, Ilina Y, Press M, Bergmann A. Sandwich immunoassay for adrenomedullin precursor and its practical application. *Sci Rep* 2024; **14**(1): 28091.
72. Riley RD, Ensor J, Snell KIE, et al. Calculating the sample size required for developing a clinical prediction model. *BMJ* 2020; **368**: m441.
73. Serra-Burriel M, Juanola A, Serra-Burriel F, et al. Development, validation, and prognostic evaluation of a risk score for long-term liver-related outcomes in the general population: a multicohort study. *Lancet* 2023; **402**(10406): 988-96.
74. Cammann VL, Würdinger M, Ghadri JR, Templin C. Takotsubo Syndrome: Uncovering Myths and Misconceptions. *Curr Atheroscler Rep* 2021; **23**(9): 53.
75. Inker LA, Eneanya ND, Coresh J, et al. New Creatinine- and Cystatin C-Based Equations to Estimate GFR without Race. *N Engl J Med* 2021; **385**(19): 1737-49.
